# Deep Reinforcement Learning for Semantic Secure Energy Efficiency Optimization in IRS-Assisted UAV Communications

**DOI:** 10.3390/s26185779

**Published:** 2026-09-11

**Authors:** Xiang Ji, Shuomin Sun, Haofei Wang, Peng Liu, Wanming Hao

**Affiliations:** 1 School of Information Engineering, Zhengzhou University, Zhengzhou 450001, China; iexiangji@zzu.edu.cn (X.J.); ssm@gs.zzu.edu.cn (S.S.); iewmhao@zzu.edu.cn (W.H.); 2 School of Aerospace and Intelligent Equipment, Xihua University, Chengdu 610039, China; 3 School of Information and Electronics, Beijing Institute of Technology, Beijing 100081, China; peng_liu@bit.edu.cn

**Keywords:** deep reinforcement learning (DRL), intelligent reflecting surface (IRS), physical-layer security, semantic communication, semantic secure energy efficiency (SSEE), unmanned aerial vehicle (UAV)

## Abstract

The integration of semantic communication with unmanned aerial vehicles (UAVs) and intelligent reflecting surfaces (IRSs) offers a promising approach for next-generation wireless systems. However, jointly optimizing semantic reliability, physical-layer security, and energy efficiency remains challenging. This paper investigates an IRS-assisted UAV semantic secure communication system in the presence of a potential eavesdropper, with the objective of maximizing semantic secure energy efficiency (SSEE). We first introduce a semantic similarity-based secure energy efficiency metric to capture the trade-off among transmission reliability, physical-layer security, and UAV energy consumption. The semantic symbol number, UAV trajectory, transmit power, and IRS phase shifts are jointly considered under mobility, secrecy, and energy constraints. To efficiently solve the resulting mixed discrete-continuous optimization problem, we develop a hierarchical optimization framework: the outer layer exhaustively searches over a finite set of candidate semantic symbol numbers, while the inner layer solves the continuous resource allocation problem using a heuristic-guided soft actor–critic (HG-SAC) algorithm. Simulation results show that the proposed framework achieves noticeable SSEE gains over several benchmark schemes.

## 1. Introduction

Sixth-generation (6G) wireless networks are evolving toward intelligent and task-oriented architectures, where communication systems must support emerging applications that demand not only efficient data transmission but also effective task execution. In applications such as unmanned aerial vehicle (UAV) cooperative control, disaster rescue, and intelligent sensing, the objective is no longer limited to accurately recovering every transmitted bit, but rather to delivering and utilizing task-related semantic information effectively. However, conventional Shannon-based communication frameworks mainly focus on bit-level reliability and cannot adequately characterize semantic relevance and task execution performance. Semantic communication fills this gap by transmitting meaningful semantic information rather than raw bits, offering a natural fit for task-oriented intelligent services. Xie et al. [1] introduced a deep learning-based semantic communication framework, namely DeepSC, which jointly optimizes semantic encoding and decoding through neural networks and demonstrates the potential of semantic-level transmission under varying channel conditions. Wang et al. [2] investigated semantic communication performance optimization by jointly considering semantic information selection and wireless resource allocation and proposed an attention-based reinforcement learning approach to maximize semantic similarity. Jiang et al. [3] further investigated wireless semantic communication for video conferencing applications and analyzed its performance under practical wireless channel conditions. Building upon these representative studies, semantic communication has attracted increasing attention for future intelligent wireless networks [4,5,6,7,8].

While semantic communication enables efficient task-oriented information transmission, its practical deployment still introduces significant challenges in wireless environments. First, semantic communication is highly sensitive to wireless channel conditions, where channel impairments may introduce semantic distortion and degrade task execution performance [9,10]. Second, protecting semantic information against unauthorized interception is equally critical. Unlike conventional bit-level communications, semantic information usually contains task-related knowledge with significantly higher application value. Once intercepted or inferred by unauthorized users, semantic information may directly reveal task intentions or decision-related content, leading to significant security risks [11,12,13]. Moreover, semantic communication requires preserving not only transmission confidentiality but also semantic fidelity for downstream tasks. Consequently, jointly guaranteeing semantic transmission reliability and physical-layer security has become a fundamental challenge in semantic communication system design. Recent work has directly tackled secure semantic communication from the perspective of semantic information protection. Li et al. [13] proposed a secure semantic communication framework that jointly considers semantic transmission quality and confidentiality. Wang et al. [14] further investigated the physical-layer security performance of semantic communication systems, highlighting the importance of security-aware transmission design. In addition, Amiriara et al. [15] demonstrated that IRS-assisted wireless systems can effectively enhance physical-layer security by intelligently reconfiguring wireless propagation environments, providing new opportunities for secure semantic communication. Besides confidentiality protection, Nan et al. [16] revealed that semantic communication systems are vulnerable to physical-layer adversarial attacks and proposed adversarial training methods to improve robustness, highlighting the importance of security-aware semantic communication design.

UAV-assisted communication offers a flexible solution for improving propagation conditions, thanks to its rapid deployment, high mobility, and strong line-of-sight (LoS) link availability. Yuan et al. [17] investigated UAV-assisted communication by jointly optimizing UAV trajectory and communication resources, demonstrating the importance of mobility control in improving communication performance. Mamaghani et al. [18] further studied joint trajectory and resource allocation optimization in UAV-assisted communication systems, showing that joint trajectory and power allocation can effectively enhance wireless transmission performance. Meanwhile, intelligent reflecting surfaces (IRSs) offer a powerful means for reconfiguring wireless propagation environments through programmable reflecting elements. Wu et al. [19] introduced IRS-assisted wireless communication and demonstrated that IRSs can improve communication performance by intelligently controlling signal propagation. For secure transmission, Hong et al. [20] investigated robust IRS-assisted secure communication under imperfect cascaded channel state information (CSI) and jointly optimized the transmit beamforming, artificial-noise covariance, and IRS phase shifts to enhance physical-layer security while reducing transmit power. Furthermore, Fang et al. [21] investigated the joint optimization of UAV trajectory and IRS phase shifts, revealing the benefits of integrating UAVs with IRSs for enhancing wireless communication performance. The integration of UAVs and IRSs provides clear benefits in improving propagation conditions, expanding wireless coverage, and supporting reliable semantic transmission in dynamic environments. However, the joint deployment of UAVs and IRSs inevitably increases propulsion and communication energy consumption, making energy-efficient resource allocation an indispensable requirement for practical implementation.

Beyond communication reliability and security, energy efficiency has become another important design objective in UAV-assisted wireless communication systems. Due to the limited onboard energy of UAVs and the joint energy consumption caused by propulsion, wireless transmission, and IRS configuration, efficient energy management is essential for practical deployment [17,22,23]. Existing studies have investigated energy-efficient resource allocation by jointly optimizing UAV trajectory, transmission resources, and IRS-related parameters. For example, Zeng et al. [23] investigated energy-efficient UAV-enabled communication and revealed the fundamental trade-off between propulsion energy consumption and communication performance. Pang et al. [22] discussed the integration of UAVs and IRSs for enhancing air–ground wireless networks, highlighting the complementary advantages of UAV mobility and IRS-enabled passive reflection. Fang et al. [21] extended this framework to IRS-assisted UAV communication systems by jointly designing UAV trajectory and IRS phase shifts. However, these optimization problems usually involve highly coupled variables, nonlinear constraints, and dynamic environments, making conventional optimization methods difficult to obtain efficient solutions with low computational complexity. Deep reinforcement learning (DRL) provides a viable path for solving such complex resource allocation problems through environment interaction. Feriani et al. [24] provided a comprehensive tutorial on applications of DRL in wireless networks and highlighted its potential for addressing dynamic resource allocation problems. Among various DRL algorithms, soft actor critic (SAC) has attracted considerable attention due to its entropy-based exploration mechanism and stable learning capability in continuous action spaces [25] and has also been applied to UAV-related optimization and control tasks, including path planning and communication optimization [26,27].

Although semantic communication, IRS-assisted UAV transmission, and intelligent resource optimization have each seen substantial progress, their integration into a unified semantic-aware UAV communication framework remains relatively limited. Most semantic communication studies concentrate on semantic representation and task-oriented delivery [1,2,3], with recent efforts further considering semantic confidentiality and semantic-aware transmission [13,14]. In parallel, IRS-assisted UAV studies have investigated the joint optimization of UAV trajectory, transmit power, and IRS phase shifts, mainly based on conventional performance metrics such as throughput, secrecy rate, and energy efficiency [21,22,27,28]. More recently, cross-layer optimization has also been introduced into secure semantic communication. For example, Wang et al. [29] proposed a cross-layer semantic secure rate (CLSSR) to jointly characterize physical-layer security and application-layer task security, and formulated its maximization as a mixed-integer nonlinear programming problem with a hierarchical reinforcement-learning-based semantic resource allocation scheme. This work provides an important foundation for cross-layer semantic security optimization, but focuses on semantic resource allocation in a general semantic communication setting without considering the coupled effects of UAV mobility, IRS-assisted propagation control, and propulsion energy consumption.

In this context, it is important to distinguish different security objectives in semantic communication. Semantic confidentiality refers to preventing unauthorized users from obtaining meaningful semantic information, whereas semantic reconstruction degradation focuses on reducing the quality or fidelity of the semantic content reconstructed by an eavesdropper. These notions differ from conventional information-theoretic secrecy, which is typically characterized by information leakage, equivocation, or secrecy capacity. Although recent studies such as [29] have demonstrated the importance of jointly considering semantic and physical-layer security. However, the joint design of semantic transmission configuration, UAV mobility, IRS-assisted propagation control, and energy consumption under a unified semantic secure energy-efficiency (SSEE) objective remains insufficiently explored. In this work, we focus on semantic confidentiality and investigate its enhancement through physical-layer security in an IRS-assisted UAV semantic communication system.

Motivated by these challenges, this paper proposes an SSEE optimization framework for IRS-assisted UAV semantic communication systems. Specifically, we first introduce an SSEE metric to jointly characterize semantic transmission reliability, physical-layer security, and energy efficiency, providing a unified performance criterion for secure semantic communication. Based on the proposed metric, a joint optimization problem is formulated by optimizing the UAV trajectory, transmit power allocation, and IRS phase shifts under dynamic wireless environments. To efficiently solve the resulting mixed discrete-continuous and non-convex optimization problem, we further develop a hierarchical optimization framework, where the outer layer determines the semantic symbol number and the inner layer employs a heuristic-guided soft actor–critic (HG-SAC) algorithm to learn effective continuous resource allocation policies for maximizing SSEE.

The main contributions of this paper are summarized as follows:

- We establish an IRS-assisted UAV semantic communication system model under dynamic wireless environments, and introduce an SSEE metric that jointly characterizes semantic transmission reliability, physical-layer security, and system energy consumption. The proposed metric provides a unified optimization objective for jointly evaluating semantic confidentiality and energy-efficient transmission.
- Based on the proposed SSEE metric, we formulate a coupled mixed discrete-continuous and non-convex optimization problem, where the semantic symbol number, UAV trajectory, transmit power allocation, and IRS phase shifts are jointly optimized to maximize SSEE under the considered security and energy constraints.
- To solve the resulting high-dimensional and non-convex problem, we propose a hierarchical HG-SAC framework. The outer layer determines the discrete semantic symbol number, while the inner layer employs HG-SAC to jointly optimize the continuous UAV trajectory, transmit power, and IRS phase shifts in the dynamic UAV-IRS environment.

The remainder of this paper is organized as follows. Section 2 presents the system model and problem formulation. Section 3 presents the proposed heuristic-guided SAC optimization framework. Section 4 provides simulation results and performance analysis. Finally, Section 5 concludes this paper.

## 2. Channel Model and Problem Formulation

In this section, the channel model, semantic communication model, and UAV energy consumption model are first established for the considered IRS-assisted UAV semantic communication system. Next, the SSEE maximization problem is formulated through the joint optimization of the UAV trajectory, transmit power, and IRS phase shifts.

### 2.1. System Model

**Figure 1:**
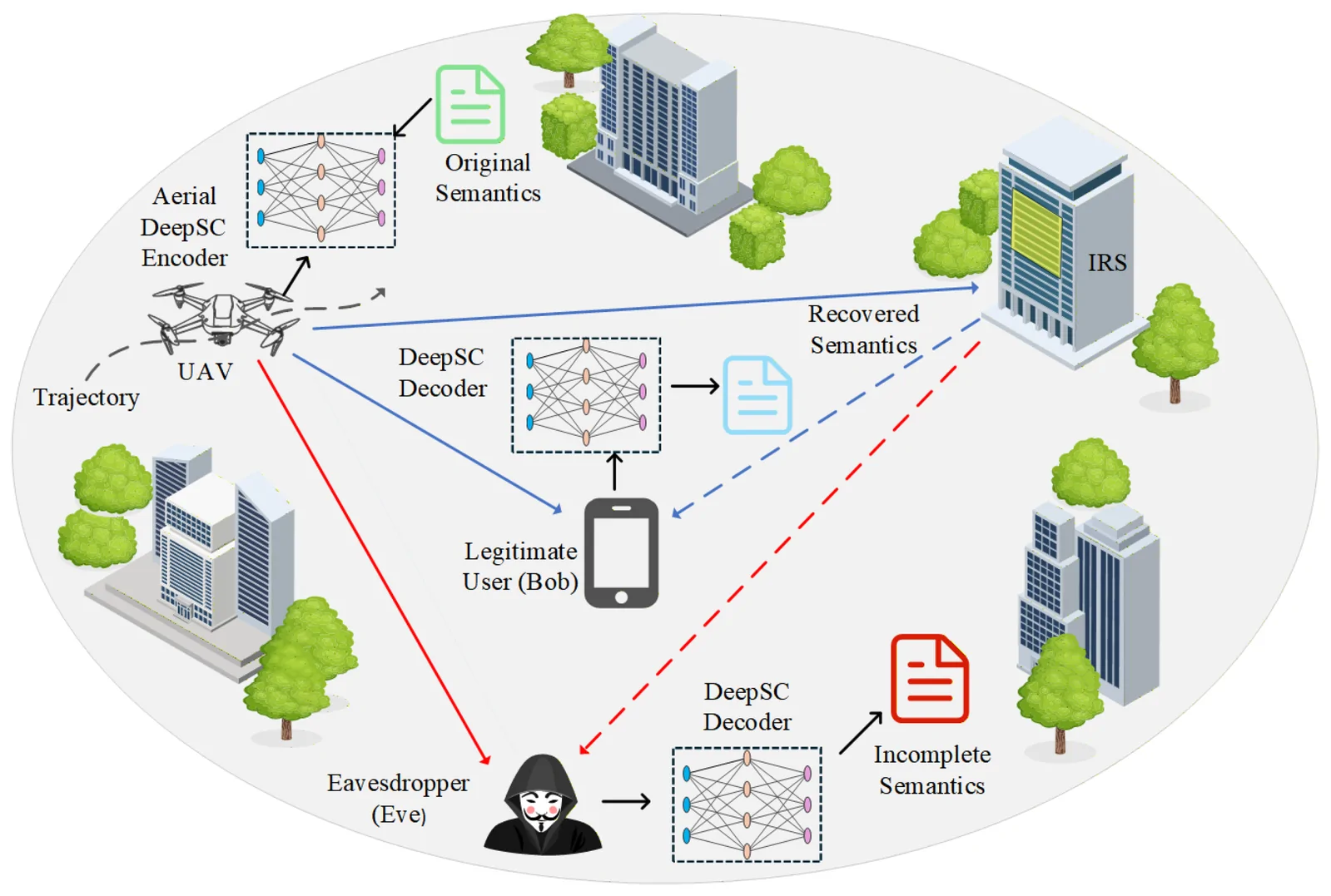
System Architecture of IRS-Assisted UAV Semantic Secure Communication.

As illustrated in Figure 1, we consider an IRS-assisted UAV semantic communication system consisting of a UAV, an IRS, a legitimate receiver (Bob), and a potential eavesdropper (Eve). The UAV acts as an aerial base station and transmits semantic information to Bob through both the direct link and the IRS-reflected link, while Eve attempts to intercept the communication. All nodes are equipped with a single antenna. To enable semantic information delivery in the considered system, DeepSC-based transceivers are adopted at both Bob and Eve. For a conservative security design, Eve is assumed to have access to the same pre-trained semantic model as Bob. The IRS is deployed to enhance the wireless propagation environment by adaptively adjusting the phase shifts in its reflecting elements. It is modeled as a uniform linear array (ULA) consisting of *M* array elements. The ULA abstraction is adopted here as a tractable one-dimensional array model for the considered system. Following the standard IRS phase shift model in IRS-assisted communications [19,21,30,31], the corresponding IRS phase shift matrix is expressed as $\Theta =\mathrm{diag}\left(e^{j\theta_{1}},e^{j\theta_{2}},\ldots ,e^{j\theta_{M}}\right),$ where $\theta_{m}\in \left[0,2\pi \right)$ denotes the phase shift in the *m*-th reflecting element for all m∈M={1,2,…,M}.

A 3D Cartesian coordinate system is employed to characterize the spatial deployment of the considered network. The positions of the IRS, Bob, and Eve are denoted by $q_{I}={\left[x_{I},y_{I},z_{I}\right]}^{T}$, $q_{B}={\left[x_{B},y_{B},z_{B}\right]}^{T}$, and $q_{E}={\left[x_{E},y_{E},z_{E}\right]}^{T}$, respectively. Following the commonly adopted UAV mobility model in UAV-assisted wireless communications [17,18], the UAV is assumed to fly at a fixed altitude $H_{0}$ over a mission duration *T*, which is discretized into *N* equal time slots with duration δ=T/N. The UAV position at time slot *n* is denoted by

(1) $$\begin{array}{c} q\left[n\right]={\left[x\left[n\right],y\left[n\right],H_{0}\right]}^{T},\;n\in \left\{1,\ldots ,N\right\}. \end{array}$$

The UAV trajectory satisfies

(2) $$\begin{array}{c} q\left[1\right]=q_{\mathrm{init}},\;q\left[N\right]=q_{\mathrm{final}}, \end{array}$$

(3) $$\begin{array}{c} \frac{∥q[n+1]-q[n]∥}{\delta }\leq V_{\max},\;\forall n, \end{array}$$

where $q_{\mathrm{init}}$ and $q_{\mathrm{final}}$ denote the initial and final UAV positions, respectively, and $V_{\max}$ is the maximum UAV speed.

In the considered dense-urban deployment, the UAV operates at a fixed altitude of $H_{0}=50$ m, whereas Bob and Eve are located at street level with $z_{B}=z_{E}=0$. The IRS is deployed at an elevated fixed location with $z_{I}=20$ m, representing an IRS mounted on an elevated urban structure.

### 2.2. Channel Model

For simplicity, this paper considers a single legitimate receiver and a single eavesdropper. Related studies have demonstrated the applicability of IRS-assisted secure wireless communication frameworks to more complex user configurations, including multi-user and multi-eavesdropper scenarios. For example, [32] investigates a DRL-based IRS-assisted secure communication framework for multiple legitimate users in the presence of multiple eavesdroppers, while [33] considers multi-user IRS-assisted secure communication through a weighted sum secrecy rate maximization objective. These studies indicate that the underlying IRS-assisted secure optimization paradigm considered in this work has the potential to be generalized to more complex user configurations. Specifically, extending the considered framework to multi-user scenarios would involve generalizing the system model and state representation to incorporate multiple legitimate receivers and eavesdroppers, as well as reformulating the reward design to account for aggregate or weighted semantic secrecy performance. Nevertheless, the present study focuses on a single legitimate receiver and a single eavesdropper, and such a generalized multi-user formulation has not been explicitly evaluated in this work. The performance of the proposed framework under more general user configurations therefore remains an important direction for future investigation.

To characterize the wireless propagation characteristics in the considered IRS-assisted UAV semantic communication system, the corresponding channel models are introduced as follows. The direct UAV–Bob and UAV–Eve links are assumed to be NLoS-dominant due to urban blockage and are therefore modeled as Rayleigh fading channels [21,28,34]. Moreover, owing to the relatively low UAV mobility and short slot duration, the channels are assumed to follow a block fading model, where the channel coefficients remain constant within each time slot while varying independently across different time slots.

Accordingly, the direct channel coefficients from the UAV to Bob and Eve at time slot *n* are expressed as

(4) $$\begin{array}{cc} h_{u,b}\left[n\right] & =\sqrt{\rho d_{u,b}^{-\alpha }\left[n\right]}{\tilde{h}}_{u,b}\left[n\right], \end{array}$$

(5) $$\begin{array}{cc} h_{u,e}\left[n\right] & =\sqrt{\rho d_{u,e}^{-\alpha }\left[n\right]}{\tilde{h}}_{u,e}\left[n\right], \end{array}$$

where ρ denotes the channel power gain at the reference distance $d_{0}=1$ m, α is the path loss exponent, $d_{u,b}\left[n\right]=∥q\left[n\right]-q_{B}∥$ and $d_{u,e}\left[n\right]=∥q\left[n\right]-q_{E}∥$ represent the UAV–Bob and UAV–Eve distances, respectively.

The IRS-assisted channels are modeled as Rician fading channels to account for the assumed dominant LoS and scattered NLoS components, following [21,30,31].

At time slot *n*, the channel vector from the UAV to the IRS is denoted by $h_{u,I}\left[n\right]\in C^{M\times 1}$, while the channel vectors from the IRS to Bob and Eve are denoted by $h_{I,b}\left[n\right]\in C^{M\times 1}$ and $h_{I,e}\left[n\right]\in C^{M\times 1}$, respectively. These channels are expressed as

(6) $$\begin{array}{cc} h_{u,I}\left[n\right] & =\sqrt{\rho d_{u,I}^{-\beta }\left[n\right]}\left(\sqrt{K/(K+1)}\;a_{u,I}\left[n\right]+\sqrt{1/(K+1)}\;{\tilde{h}}_{u,I}^{\mathrm{NLoS}}\left[n\right]\right), \end{array}$$

(7) $$\begin{array}{cc} h_{I,b}\left[n\right] & =\sqrt{\rho d_{I,b}^{-\beta }\left[n\right]}\left(\sqrt{K/(K+1)}\;a_{I,b}\left[n\right]+\sqrt{1/(K+1)}\;{\tilde{h}}_{I,b}^{\mathrm{NLoS}}\left[n\right]\right), \end{array}$$

(8) $$\begin{array}{cc} h_{I,e}\left[n\right] & =\sqrt{\rho d_{I,e}^{-\beta }\left[n\right]}\left(\sqrt{K/(K+1)}\;a_{I,e}\left[n\right]+\sqrt{1/(K+1)}\;{\tilde{h}}_{I,e}^{\mathrm{NLoS}}\left[n\right]\right), \end{array}$$

where β denotes the path loss exponent and *K* is the Rician factor. For the UAV–IRS, IRS–Bob, and IRS–Eve links, a common Rician *K*-factor is adopted to characterize a dominant specular component and scattered multipath components. Although the three links may experience different propagation conditions in practical deployments, the common *K*-factor is used as a simplified and consistent channel-statistical assumption, thereby avoiding additional link-dependent channel parameters. The distances of the UAV–IRS, IRS–Bob, and IRS–Eve links are denoted by $d_{u,I}\left[n\right]$, $d_{I,b}\left[n\right]$, and $d_{I,e}\left[n\right]$, respectively.

The vectors $a_{u,I}\left[n\right]$, $a_{I,b}\left[n\right]$, and $a_{I,e}\left[n\right]$ represent the ULA array responses of the LoS components, given by

(9) $$\begin{array}{cc} a_{u,I}\left[n\right] & ={\left[1,e^{-j\frac{2\pi }{\lambda }d\phi_{u,I}\left[n\right]},\ldots ,e^{-j\frac{2\pi }{\lambda }\left(M-1\right)d\phi_{u,I}\left[n\right]}\right]}^{T}, \end{array}$$

(10) $$\begin{array}{cc} a_{I,b}\left[n\right] & ={\left[1,e^{-j\frac{2\pi }{\lambda }d\phi_{I,b}\left[n\right]},\ldots ,e^{-j\frac{2\pi }{\lambda }\left(M-1\right)d\phi_{I,b}\left[n\right]}\right]}^{T}, \end{array}$$

(11) $$\begin{array}{cc} a_{I,e}\left[n\right] & ={\left[1,e^{-j\frac{2\pi }{\lambda }d\phi_{I,e}\left[n\right]},\ldots ,e^{-j\frac{2\pi }{\lambda }\left(M-1\right)d\phi_{I,e}\left[n\right]}\right]}^{T}, \end{array}$$

where $\phi_{u,I}\left[n\right]$, $\phi_{I,b}\left[n\right]$, and $\phi_{I,e}\left[n\right]$ denote the corresponding direction cosines with respect to the ULA axis, *j* denotes the imaginary unit, λ is the carrier wavelength, and *d* is the IRS inter-element spacing. Moreover, ${\tilde{h}}_{u,I}^{\mathrm{NLoS}}\left[n\right]$, ${\tilde{h}}_{I,b}^{\mathrm{NLoS}}\left[n\right]$, and ${\tilde{h}}_{I,e}^{\mathrm{NLoS}}\left[n\right]$ are modeled as independent zero-mean unit-variance complex Gaussian random vectors [21,30]. For analytical tractability, perfect CSI is assumed for both the legitimate communication links and the eavesdropping links, following the widely adopted assumption in IRS-assisted secure communication studies [18,21,28]. This assumption is idealized, as practical UAV-assisted wireless systems inevitably suffer from channel estimation errors and other CSI imperfections. Consequently, the performance obtained under perfect CSI should be interpreted as an optimistic reference for practical system performance. Accordingly, the composite channels from the UAV to Bob and Eve are expressed as

(12) $$\begin{array}{cc} h_{b}\left[n\right] & =h_{u,b}\left[n\right]+h_{I,b}^{H}\left[n\right]\Theta \left[n\right]h_{u,I}\left[n\right], \end{array}$$

(13) $$\begin{array}{cc} h_{e}\left[n\right] & =h_{u,e}\left[n\right]+h_{I,e}^{H}\left[n\right]\Theta \left[n\right]h_{u,I}\left[n\right], \end{array}$$

The received signals at Bob and Eve are given by

(14) $$\begin{array}{cc} y_{b}\left[n\right] & =h_{b}\left[n\right]x\left[n\right]+n_{b}\left[n\right], \end{array}$$

(15) $$\begin{array}{cc} y_{e}\left[n\right] & =h_{e}\left[n\right]x\left[n\right]+n_{e}\left[n\right], \end{array}$$

where $n_{b}\left[n\right]$ and $n_{e}\left[n\right]$ denote additive white Gaussian noise (AWGN) at Bob and Eve, respectively. Accordingly, the received SNRs at Bob and Eve are expressed as

(16) $$\begin{array}{cc} \gamma_{b}\left[n\right] & =\frac{P\left[n\right]|h_{b}{\left[n\right]|}^{2}}{\sigma_{b}^{2}}, \end{array}$$

(17) $$\begin{array}{cc} \gamma_{e}\left[n\right] & =\frac{P\left[n\right]|h_{e}{\left[n\right]|}^{2}}{\sigma_{e}^{2}}, \end{array}$$

where $\sigma_{b}^{2}$ and $\sigma_{e}^{2}$ denote the noise powers at Bob and Eve, respectively.

### 2.3. Semantic Communication Model

Unlike conventional communication systems that rely on bit-level transmission metrics, semantic information in the considered system is conveyed through semantic symbols instead of conventional bit sequences. According to [1,35], the performance of semantic communication in text transmission can be evaluated through a performance metric called semantic similarity, which can be expressed as follows

(18) $$\begin{array}{c} \xi =\frac{B{\left(s\right)}^{⊤}B\left(\hat{s}\right)}{\left\|B(s)\|\;\|B(\hat{s})\right\|}, \end{array}$$

where B(·) denotes the sentence embedding obtained from a pre-trained Sentence-BERT model [36], s is the transmitted sentence, and $\hat{s}$ is the reconstructed sentence after channel transmission and semantic decoding. The semantic similarity satisfies 0≤ξ≤1, where a larger value indicates better semantic reconstruction performance.

Since conventional bit-rate metrics cannot adequately characterize semantic communication quality, the semantic rate (S-R) is adopted to quantify the effective semantic information delivered per unit time. Following [37], the semantic rates at Bob and Eve are defined as

(19) $$\begin{array}{c} S_{B}=\frac{WI}{N_{s}L}\xi_{B},\;S_{E}=\frac{WI}{N_{s}L}\xi_{E}, \end{array}$$

where *W* denotes the channel bandwidth in Hz and is interpreted as the available symbol transmission rate in symbols/s under the adopted system model, *I* denotes the expected semantic information contained in each sentence, measured in suts/sentence, $N_{s}$ denotes the average number of semantic symbols allocated to represent each word, measured in symbols/word, and *L* denotes the expected number of words per sentence, measured in words/sentence. Since $N_{s}L$ represents the average number of semantic symbols required to transmit one sentence, $W/(N_{s}L)$ gives the sentence transmission rate in sentences/s. Therefore, multiplying it by *I* and the dimensionless semantic similarity ξ yields the semantic rate in suts/s.

It can be observed from the above semantic rate formulation that the semantic communication performance is directly affected by the semantic similarity. In DeepSC-based systems, semantic similarity is jointly influenced by the semantic symbol allocation $N_{s}$ and the received signal-to-noise ratio (SNR) γ. However, the exact analytical relationship among semantic similarity, semantic symbol allocation, and channel quality depends on the adopted neural semantic communication architecture and is generally unavailable in closed form due to the nonlinear end-to-end mapping introduced by deep neural networks. Therefore, in the absence of an explicit analytical expression relating semantic similarity to the received SNR and semantic symbol allocation, we obtain the semantic performance data by running the open-source implementation of DeepSC [1] and construct a lookup table to characterize semantic transmission performance. Let $\xi (N_{s},\gamma )$ denote the semantic similarity obtained from the adopted DeepSC-based lookup table for a given semantic symbol allocation $N_{s}$ and received SNR γ. For operating points whose received SNR values do not exactly coincide with the tabulated entries, linear interpolation is employed to estimate the corresponding semantic similarity.

**Figure 2:**
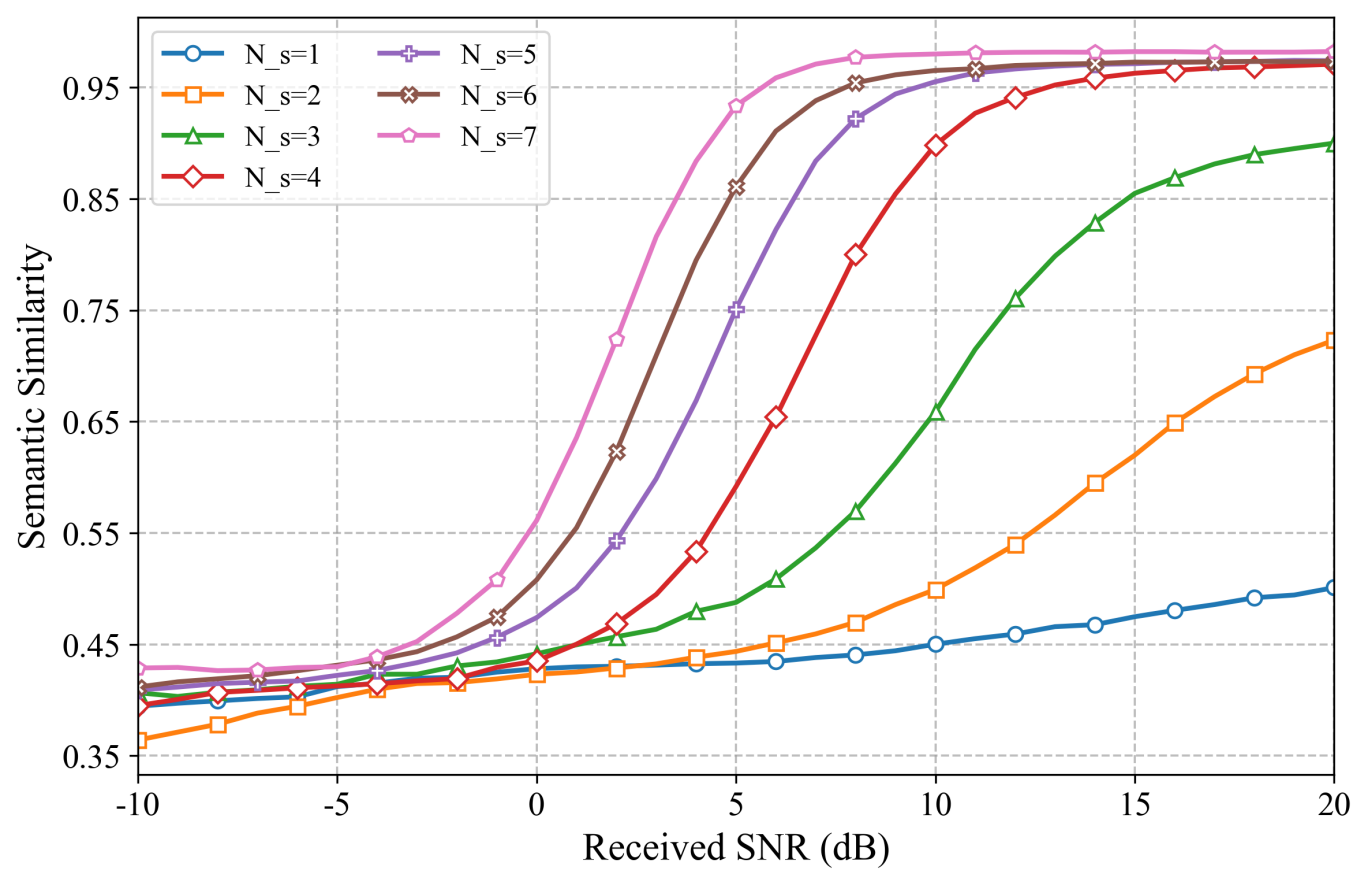
Semantic similarity versus received SNR under different semantic symbol allocations based on the DeepSC lookup-table data [1].

Figure 2 shows the semantic similarity versus the received SNR under different semantic symbol allocations based on the DeepSC lookup-table data. It can be observed that the semantic similarity generally increases with the received SNR. In addition, for the adopted DeepSC configuration, allocating more semantic symbols generally improves semantic reconstruction performance over the considered operating range. Hence, the adopted lookup-table-based semantic characterization provides a data-driven basis for evaluating the semantic transmission performance in the subsequent optimization process [1,35].

It should be noted that the semantic performance characterization in this work relies on the pretrained DeepSC model, which is a text-oriented semantic communication architecture. Accordingly, the specific semantic similarity characteristics and quantitative SSEE results reported in this paper depend on the adopted DeepSC encoder–decoder configuration. Using a different semantic communication architecture may lead to different semantic performance characteristics, optimal semantic symbol allocations, and numerical SSEE results. Nevertheless, the proposed hierarchical HG-SAC framework does not fundamentally rely on the specific DeepSC architecture. In the optimization process, the semantic communication model serves as a performance evaluation module, and researchers can replace the corresponding semantic performance characterization when adopting a different encoder–decoder. Therefore, the hierarchical HG-SAC optimization framework can be generally extended to different semantic communication architectures.

### 2.4. Energy Consumption Model

The total energy consumption of the UAV consists of propulsion energy and communication-related energy [17,23]. Among these components, propulsion energy usually dominates the overall UAV energy consumption during flight. In the considered rotary-wing UAV system, the propulsion power at time slot *n* with horizontal velocity v[n] is modeled as [38]

(20) $$\begin{array}{cc} P_{\mathrm{fly}}\left[n\right] & =P_{0}\left(1+\frac{3v^{2}\left[n\right]}{U_{\mathrm{tip}}^{2}}\right)\\ \; & \;+P_{i}{\left(\sqrt{1+\frac{v^{4}\left[n\right]}{4v_{0}^{4}}}-\frac{v^{2}\left[n\right]}{2v_{0}^{2}}\right)}^{\frac{1}{2}}\\ \; & \;+\frac{1}{2}d_{0}\rho_{\mathrm{air}}sAv^{3}\left[n\right] \end{array}$$

where $P_{0}$ denotes the blade profile power, $P_{i}$ denotes the induced power in hovering, $U_{\mathrm{tip}}$ denotes the rotor blade tip speed, $v_{0}$ denotes the mean rotor-induced velocity in hovering, $d_{0}$ denotes the fuselage drag ratio, $\rho_{\mathrm{air}}$ denotes the air density, *s* denotes the rotor solidity, and *A* denotes the rotor disc area. Accordingly, the propulsion energy consumption over one time slot with duration δ is given by

(21) $$\begin{array}{c} E_{\mathrm{fly}}\left[n\right]=\delta P_{\mathrm{fly}}\left[n\right], \end{array}$$

In addition to the propulsion energy, the communication-related energy consumption is given by

(22) $$\begin{array}{c} E_{\mathrm{comm}}\left[n\right]=\delta P\left[n\right], \end{array}$$

where P[n] denotes the RF transmit power delivered to the UAV’s antenna in time slot *n*. For simplicity, the power amplifier (PA) inefficiency is not explicitly considered, and P[n] is directly used to characterize the communication-related energy consumption. Circuit power and other hardware-related energy consumption are also neglected. Therefore, the total energy consumption of the UAV over the entire mission period is expressed as

(23) $$\begin{array}{c} E_{\mathrm{total}}=\sum_{n=1}^{N}\left(E_{\mathrm{fly}}\left[n\right]+E_{\mathrm{comm}}\left[n\right]\right). \end{array}$$

### 2.5. Optimization Problem Formulation

Based on the established channel, semantic communication, and energy consumption models, we formulate the overall SSEE maximization problem by considering the semantic symbol number $N_{s}$ as a discrete optimization variable in the outer layer, while jointly optimizing the UAV trajectory, transmit power, and IRS phase shifts as continuous variables in the inner layer. Following [37], the semantic secure rate at time slot *n* is defined as

(24) $$\begin{array}{c} S_{\sec}\left[n\right]={\left[S_{B}\left[n\right]-S_{E}\left[n\right]\right]}^{+}, \end{array}$$

where ${\left[x\right]}^{+}=\max\left(x,0\right)$, $S_{\sec}\left[n\right]$ denotes the semantic secure transmission rate (in suts/s), and $\delta S_{\sec}\left[n\right]$ represents the amount of securely transmitted semantic information in time slot *n*. This definition follows the semantic-level security characterization, where the difference between the semantic performance of the legitimate user and that of the eavesdropper is used to quantify the security advantage. In the considered system, $S_{B}\left[n\right]$ and $S_{E}\left[n\right]$ measure the effective semantic information delivery rates at Bob and Eve, respectively. Therefore, their positive difference quantifies the semantic information delivery advantage of Bob over Eve. The positive part operator further ensures that no positive semantic security advantage is assigned when Eve achieves an equal or higher semantic rate. Accordingly, the SSEE is defined as the amount of securely delivered semantic information per unit energy consumption, which can be expressed as

(25) $$\begin{array}{cc} \; & \max_{N_{s}\in N_{s}}\{\max_{q[n],\;P[n],\;\Theta [n]}\frac{\sum_{n=1}^{N}\delta S_{sec}\left[n\right]}{E_{\mathrm{total}}}\} \end{array}$$

(25a) $$\begin{array}{cc} s.t.\; & ∥q\left[n+1\right]-q\left[n\right]∥\leq V_{\max}\delta ,\;\forall n \end{array}$$

(25b) $$\begin{array}{cc} & q[n]\in A,\;\forall n\;\;\;\;\;\; \end{array}$$

(25c) $$\begin{array}{cc} & q\left[1\right]=q_{init},\;q\left[N\right]=q_{final} \end{array}$$

(25d) $$\begin{array}{cc} & \;\;\;\;\;0\leq P\left[n\right]\leq P_{\max},\;\forall n\;\;\;\; \end{array}$$

(25e) $$\begin{array}{cc} & \;\;\;\;\;0\leq \theta_{m}\left[n\right]\leq 2\pi ,\;\forall m,n\;\;\;\; \end{array}$$

(25f) $$\begin{array}{cc} & \;\;\;\;\;\;\;\sum_{n=1}^{N}\left(E_{fly}\left[n\right]+E_{\mathrm{comm}}\left[n\right]\right)\leq E_{\max} \end{array}$$

(25g) $$\begin{array}{cc} & \;\;\;\;\;\;\;\frac{1}{N}\sum_{n=1}^{N}S_{sec}\left[n\right]\geq S_{\min}\;\;\;\;\; \end{array}$$

where $N_{s}$ denotes the finite set of candidate semantic symbol numbers. The outer maximization selects the semantic symbol number $N_{s}$, while the inner maximization optimizes the continuous control variables q[n], P[n], and Θ[n] for each fixed $N_{s}$, subject to constraints (25a)–(25g). Constraints (25a)–(25c) describe the UAV mobility, including velocity limitation, feasible flight region, and trajectory boundary conditions. Constraints (25d) and (25e) impose the transmit power budget and IRS phase shift restrictions, respectively. Constraint (25f) guarantees the total energy budget of the UAV, accounting for both propulsion and communication energy consumption. Constraint (25g) ensures the minimum semantic secure rate requirement to maintain reliable and secure communications.

## 3. DRL-Based Optimization for Semantic Secure Energy Efficiency

Given the strong coupling and dynamic environment discussed above, DRL provides a more suitable analytical framework, and among DRL algorithms, SAC is selected for its ability to handle continuous action spaces while ensuring training stability through entropy regularization. The formulated optimization framework is highly challenging due to the coexistence of continuous resource allocation variables and a discrete semantic symbol configuration. As a result, problem 25 is a mixed discrete-continuous optimization problem. While conventional methods such as alternating optimization (AO), successive convex approximation (SCA), and block coordinate descent (BCD) are in principle applicable, they typically suffer from high computational complexity and slow convergence because of the problem’s non-convexity and strong variable coupling.

### 3.1. Hierarchical Optimization and Computational Complexity

The optimization problem in (25) is a mixed discrete-continuous non-convex optimization problem, where $N_{s}$ is a discrete variable and the UAV trajectory, transmit power, and IRS phase shifts are continuous variables. To avoid directly handling the discrete and high-dimensional continuous variables within a unified optimization procedure, a hierarchical optimization strategy is adopted. Specifically, $N_{s}$ is exhaustively searched over its finite candidate set, while the remaining continuous variables are jointly optimized by the proposed HG-SAC algorithm.

Let $N_{s}=\left\{1,2,\ldots ,N_{s}^{\max}\right\}$ denote the candidate set of $N_{s}$. For each fixed $N_{s}$, the original problem is reduced to a continuous non-convex optimization problem. Thus, the discrete layer does not introduce an approximation, since all feasible values of $N_{s}$ are explicitly evaluated. In particular, if the continuous subproblem for each fixed $N_{s}$ were solved globally, exhaustive enumeration would preserve the global optimum of the original mixed discrete-continuous problem. In practice, HG-SAC provides an efficient approximate solution to the continuous subproblem.

The computational complexity of the proposed hierarchical framework consists primarily of the HG-SAC optimization of the continuous variables and the finite enumeration of $N_{s}$. If $C_{\mathrm{HG}\text{-}\mathrm{SAC}}$ denotes the computational cost of training HG-SAC for one fixed $N_{s}$, the overall complexity can be expressed as

(26) $$C_{\mathrm{hier}}=O\left(|N_{s}|C_{\mathrm{HG}\text{-}\mathrm{SAC}}\right).$$

Since $N_{s}\in \left\{1,\ldots ,7\right\}$ in this work, the outer exhaustive search involves only seven candidate values and therefore introduces a small finite computational overhead.

For comparison, an AO method would require repeatedly solving separate subproblems for the UAV trajectory, transmit power, and IRS phase shifts until convergence. The computational burden therefore increases with the number of AO iterations and the dimensions of the coupled variable blocks. A branch-and-bound (B&B) method could provide a global-search framework, but its worst-case complexity generally grows exponentially with the dimension of the continuous decision space. In contrast, the proposed HG-SAC directly learns the joint control policy for the continuous variables and avoids repeatedly solving high-dimensional non-convex subproblems or performing global partitioning. Therefore, the proposed hierarchical scheme exhaustively evaluates the finite discrete variable while efficiently optimizing the continuous variables, providing a favorable trade-off between search completeness and computational complexity.

For each fixed $N_{s}$, the resulting continuous optimization problem is formulated as a POMDP, as described below.

### 3.2. POMDP Formulation

To facilitate DRL-based optimization, the formulated long-term SSEE maximization problem is modeled as a POMDP, where the IRS-assisted UAV semantic communication system serves as the environment and the UAV controller acts as the intelligent agent. The considered POMDP consists of the observable state space S, action space A, transition distribution T, and reward function R. At each time slot, the agent observes the available system information, selects an action, receives the corresponding reward from the environment, and proceeds to the next time slot. The key components of the formulated POMDP are described as follows.

Observable state space: Let S denote the observable state space available to the UAV controller. The observable state at time slot *n* is defined as

(27) $$\begin{array}{c} s\left[n\right]\in S=\left[{\tilde{t}}_{n},\tilde{q}\left[n\right],{\tilde{E}}_{c}\left[n\right],{\tilde{S}}_{sec}\left[n-1\right]\right], \end{array}$$

where ${\tilde{t}}_{n}$ denotes the normalized time index, $\tilde{q}\left[n\right]$ represents the normalized UAV position, ${\tilde{E}}_{c}\left[n\right]$ denotes the normalized cumulative consumed energy, and ${\tilde{S}}_{sec}\left[n-1\right]$ denotes the normalized semantic secure transmission performance observed in the previous time slot. The normalization factors in (27) are specified as ${\tilde{t}}_{n}=\frac{n-1}{N-1}$, $\tilde{q}\left[n\right]={\left[2\frac{x\left[n\right]-x_{\min}}{x_{\max}-x_{\min}}-1,\;2\frac{y\left[n\right]-y_{\min}}{y_{\max}-y_{\min}}-1,\;\frac{z[n]}{H}\right]}^{⊤}$, ${\tilde{E}}_{c}\left[n\right]=\frac{E_{c}\left[n\right]}{E_{\max}}$, and $E_{c}\left[n\right]=\sum_{i=1}^{n-1}\left(E_{\mathrm{fly}}\left[i\right]+E_{\mathrm{comm}}\left[i\right]\right)$. Here, *N* is the total number of mission slots, $\left[x_{\min},x_{\max}\right]$ and $\left[y_{\min},y_{\max}\right]$ denote the horizontal deployment region, *H* is the fixed UAV altitude, and $E_{\max}$ is the total mission energy budget.

The observable state is designed as a compact representation of the system information available to the UAV controller. The UAV position captures the network geometry and large-scale channel variations, while the small-scale fading is generated by the environment according to the adopted block-fading channel model. The instantaneous channel coefficients are not explicitly included in the observable state to reduce the observation dimension and avoid requiring instantaneous CSI to be explicitly acquired, fed back, and incorporated as an input to the online policy.

Action space: Let A denote the action space. The action executed by the agent at time slot *n* is defined as

(28) $$\begin{array}{c} a\left[n\right]\in A=[\Delta q[n],P[n],\theta [n]], \end{array}$$

where Δq[n] denotes the UAV trajectory update, P[n] denotes the UAV transmit power, and $\theta \left[n\right]={\left[\theta_{1}\left[n\right],\ldots ,\theta_{M}\left[n\right]\right]}^{T}$ represents the IRS phase-shift vector, where $\theta_{m}\left[n\right]\in \left[0,2\pi \right)$ for m=1,…,M. Since Δq[n] contains two horizontal displacement variables, and P[n] is a scalar transmit-power variable, the action vector has a total dimension of M+3. The IRS phase shifts remain directly controllable through the action vector, while their values are generated by the learned policy based on the available observable state rather than through explicit instantaneous-CSI-based phase optimization at each time slot.

The SAC actor outputs a normalized action vector

(29) $$\tilde{a}\left[n\right]={\left[a_{x}\left[n\right],a_{y}\left[n\right],a_{p}\left[n\right],a_{\theta ,1}\left[n\right],\ldots ,a_{\theta ,M}\left[n\right]\right]}^{⊤},$$

where all action components are bounded in [−1,1] by the final tanh(·) activation. Here, $a_{x}\left[n\right]$ and $a_{y}\left[n\right]$ denote the normalized horizontal UAV displacement actions along the *x*- and *y*-axes, respectively, $a_{p}\left[n\right]$ denotes the normalized transmit-power control action, and $a_{\theta ,m}\left[n\right]$ denotes the normalized phase-control action for the *m*-th IRS element. The normalized power action is linearly mapped to the feasible transmit-power range as $P\left[n\right]=\left(a_{p}\left[n\right]+1\right)P_{\max}/2$, which ensures $P\left[n\right]\in \left[0,P_{\max}\right]$. Similarly, the normalized phase action is mapped as $\theta_{m}\left[n\right]=\pi \left(a_{\theta ,m}\left[n\right]+1\right)$ for m=1,…,M. For UAV motion, the normalized displacement actions are scaled according to the maximum UAV speed and slot duration as $\Delta x\left[n\right]=a_{x}\left[n\right]V_{\max}\delta$ and $\Delta y\left[n\right]=a_{y}\left[n\right]V_{\max}\delta$, respectively. The corresponding candidate UAV position is $\tilde{q}\left[n+1\right]=q\left[n\right]+{\left[\Delta x\left[n\right],\Delta y\left[n\right],0\right]}^{⊤}$. The candidate position is subsequently adjusted to satisfy the UAV mobility constraints defined in Section 2, including the maximum-speed, flight-region, and terminal-reachability constraints.

Observable state transition: Let T(s[n+1]|s[n],a[n]) denote the transition distribution of the observable state from the current state s[n] to the next state s[n+1] after executing action a[n].

Reward function: The original optimization objective aims to maximize the SSEE over the entire mission horizon. Therefore, the terminal reward is designed to directly reflect the long-term SSEE objective, which is defined as

(30) $$\begin{array}{c} r_{\mathrm{ter}}=\frac{\sum_{n=1}^{N}\delta S_{\sec}\left[n\right]}{\sum_{n=1}^{N}\left(E_{\mathrm{fly}}\left[n\right]+E_{\mathrm{comm}}\left[n\right]\right)}. \end{array}$$

Here, $r_{\mathrm{ter}}$ is calculated after completing the entire mission and is exactly consistent with the original SSEE definition. However, directly using only the terminal reward leads to sparse feedback during training, which may degrade the learning efficiency of the DRL agent. Therefore, an instantaneous reward is further introduced as an auxiliary dense learning signal, given by

(31) $$\begin{array}{c} r_{\mathrm{ins}}\left[n\right]=\frac{\delta S_{\sec}\left[n\right]}{E_{\mathrm{fly}}\left[n\right]+E_{\mathrm{comm}}\left[n\right]}, \end{array}$$

where, $\delta S_{\sec}\left[n\right]$ represents the securely transmitted semantic information during time slot *n*, while $E_{\mathrm{fly}}\left[n\right]$ and $E_{\mathrm{comm}}\left[n\right]$ denote the UAV propulsion energy consumption and communication energy consumption, respectively.

It is important to note that the cumulative instantaneous reward is not mathematically equivalent to the original episode-level SSEE objective, since, in general,

(32) $$\begin{array}{c} \sum_{n=1}^{N}\frac{\delta S_{\sec}\left[n\right]}{E_{\mathrm{fly}}\left[n\right]+E_{\mathrm{comm}}\left[n\right]}\neq \frac{\sum_{n=1}^{N}\delta S_{\sec}\left[n\right]}{\sum_{n=1}^{N}\left(E_{\mathrm{fly}}\left[n\right]+E_{\mathrm{comm}}\left[n\right]\right)}. \end{array}$$

Therefore, the instantaneous reward is used as an auxiliary dense learning signal to facilitate policy training, rather than as a replacement for the original episode-level SSEE objective.

Accordingly, the reward at time slot *n* is designed as

(33) $$\begin{array}{c} r\left[n\right]=I\left(n=N\right)r_{\mathrm{ter}}+r_{\mathrm{ins}}\left[n\right]-R_{\mathrm{penalty}}\left[n\right], \end{array}$$

where I(n=N) is an indicator function that equals one only at the end of the mission episode. Moreover, $R_{\mathrm{penalty}}\left[n\right]$ denotes the penalty imposed when the current state-action transition violates the prescribed system constraints, including energy-budget, semantic secure transmission, and terminal-reachability constraints.

The penalty term in (33) is explicitly defined as

(34) $$\begin{array}{cc} R_{\mathrm{penalty}}\left[n\right] & =\lambda_{E}{\left[\frac{E_{c}\left[n\right]+E_{\mathrm{fly}}\left[n\right]+E_{\mathrm{comm}}\left[n\right]-E_{\max}}{E_{\max}}\right]}^{+}\\ \; & \;+\lambda_{S}I\;\left(S_{\sec}\left[n\right]<S_{\min}\right)+\lambda_{U}I\;\left(d_{F}\left[n+1\right]>N_{\mathrm{rem}}\left[n\right]V_{\max}\delta \right), \end{array}$$

where ${\left[x\right]}^{+}=\max\left\{x,0\right\}$, $d_{F}\left[n+1\right]$ denotes the distance from the updated UAV position to the final destination and $N_{\mathrm{rem}}\left[n\right]=N-n$ denotes the number of remaining time slots after slot *n*. The coefficients $\lambda_{E}$, $\lambda_{S}$, and $\lambda_{U}$ control the penalties associated with energy-budget violation, insufficient slot-wise semantic secure transmission performance, and terminal-unreachability, respectively.

Accordingly, the HG-SAC policy is trained by maximizing the expected cumulative discounted training reward, given by

(35) $$\begin{array}{c} J_{\mathrm{train}}\left(\pi \right)=E_{\pi }\left[\sum_{n=1}^{N}\gamma^{n-1}r\left[n\right]\right], \end{array}$$

where γ∈(0,1) denotes the discount factor. Since the training reward combines the terminal objective-related component with the auxiliary instantaneous reward and constraint-related penalties, $J_{\mathrm{train}}\left(\pi \right)$ serves as a surrogate training objective to facilitate efficient policy learning. Accordingly, the SAC optimization is performed with respect to this surrogate training objective, while the original episode-level SSEE remains the ultimate performance metric for evaluating the learned policy.

### 3.3. HG-SAC Learning Framework

**Figure 3:**
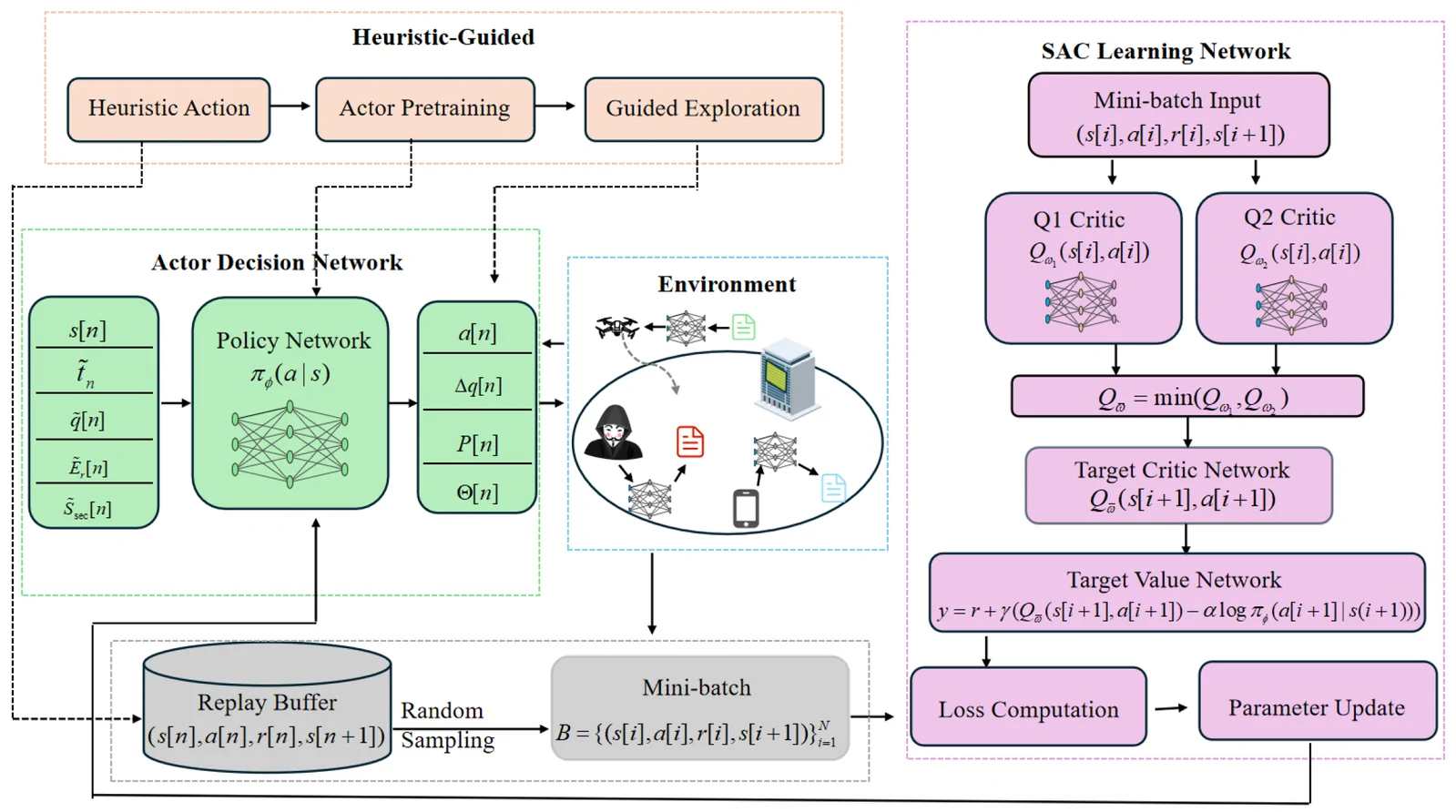
HG-SAC Framework for SSEE Optimization.

To solve the above POMDP, a HG-SAC framework is adopted, as illustrated in Figure 3.

In line with the SAC algorithm, the proposed framework optimizes both the expected cumulative return and the policy entropy to encourage exploration and improve training robustness. The objective function is given by

(36) $$\begin{array}{c} J_{\mathrm{SAC}}=E_{\pi }\left[\sum_{n=1}^{N}\gamma^{n-1}\left(r[n]+\alpha H(\pi (\cdot |s[n]))\right)\right], \end{array}$$

where α denotes the entropy temperature coefficient, and H(π(·|s[n])) represents the policy entropy under state s[n]. Here, r[n] is the training reward defined in the POMDP formulation, which combines the auxiliary instantaneous reward with the terminal episode-level SSEE reward and constraint penalties. The original episode-level SSEE remains the performance metric used to evaluate the learned policy.

To improve training stability and alleviate the overestimation bias, twin critic networks are adopted in the SAC framework. The target Q-value for SAC training is computed as

(37) $$\begin{array}{c} y\left[n\right]=r\left[n\right]+\gamma (\min_{i=1,2}Q_{{\bar{\omega }}_{i}}(s\left[n+1\right],a\left[n+1\right])\\ -\alpha \log\pi (a[n+1]|s[n+1])), \end{array}$$

where $Q_{{\bar{\omega }}_{i}}\left(\cdot \right)$ denotes the *i*-th critic network parameterized by ${\bar{\omega }}_{i}$. The critic networks are trained by minimizing the mean squared Bellman error between the predicted Q-value and the target Q-value. Specifically, the critic loss function is given by

(38) $$\begin{array}{c} L_{Q}\left(\omega_{i}\right)=E_{(s,a,r,s^{\prime })∼D}\left[{\left(Q_{\omega_{i}}\left(s,a\right)-y\left[n\right]\right)}^{2}\right]\;i\in \left\{1,2\right\}, \end{array}$$

where D denotes the replay buffer. Meanwhile, the actor network is updated by maximizing the expected soft Q-value together with the entropy regularization term. Accordingly, the actor loss function can be expressed as

(39) $$\begin{array}{c} L_{\pi }\left(\phi \right)=E_{s∼D}\left[\alpha \log\pi_{\phi }\left(a|s\right)-\min_{i=1,2}Q_{\omega_{i}}\left(s,a\right)\right], \end{array}$$

where ϕ denotes the parameters of the actor network. To improve training stability, soft target network updates are adopted according to

(40) $$\begin{array}{c} {\bar{\omega }}_{i}\leftarrow \tau \omega_{i}+\left(1-\tau \right){\bar{\omega }}_{i},\;i\in \left\{1,2\right\}, \end{array}$$

where ${\bar{\omega }}_{i}$ denotes the parameters of the target critic network and τ∈(0,1) represents the soft update coefficient.

To improve convergence efficiency and exploration performance, a heuristic-guided policy is incorporated into the SAC framework. Let

(41) $$\begin{array}{c} a_{h}\left[n\right]=\pi_{h}\left(s\left[n\right]\right) \end{array}$$

denote the heuristic action generated by the heuristic policy $\pi_{h}\left(\cdot \right)$. Specifically,

(42) $$\begin{array}{c} a_{h}\left[n\right]={\left[a_{x,h}\left[n\right],a_{y,h}\left[n\right],a_{p,h}\left[n\right],a_{\theta ,1,h}\left[n\right],\ldots ,a_{\theta ,M,h}\left[n\right]\right]}^{⊤}, \end{array}$$

where the components correspond to the normalized UAV displacement, transmit power, and IRS phase-shift actions, respectively.

For UAV trajectory generation, the heuristic policy adopts a two-stage waypoint-based rule. In the first stage, the UAV moves toward a predefined intermediate waypoint, while in the second stage, it proceeds toward the final destination. At each time slot, the remaining displacement toward the target point is distributed over the remaining time slots of the current stage and normalized by the maximum allowable displacement. Specifically,

(43) $$\begin{array}{cc} a_{x,h}\left[n\right]\; & =\mathrm{clip}\left(\frac{x_{n}^{\mathrm{tar}}-x_{n}}{N_{n}\Delta_{\max}},-1,1\right),\\ a_{y,h}\left[n\right]\; & =\mathrm{clip}\left(\frac{y_{n}^{\mathrm{tar}}-y_{n}}{N_{n}\Delta_{\max}},-1,1\right), \end{array}$$

where $q_{n}^{\mathrm{tar}}={\left[x_{n}^{\mathrm{tar}},y_{n}^{\mathrm{tar}},H\right]}^{⊤}$ denotes the waypoint or final destination selected according to the current flight stage, $N_{n}$ denotes the remaining number of time slots in that stage, and $\Delta_{\max}=V_{\max}\delta$ is the maximum horizontal displacement per time slot. The clipping operation confines the normalized displacement actions to [−1,1].

For transmit-power control, the heuristic policy uses a geometry-based Bob–Eve advantage metric

(44) $$\eta_{n}=\frac{d_{E,n}-d_{B,n}}{D_{\max}},$$

where $d_{B,n}$ and $d_{E,n}$ denote the UAV-to-Bob and UAV-to-Eve distances, respectively. The normalized transmit-power ratio $\rho_{n}$, representing the fraction of the maximum transmit power $P_{\max}$ used by the heuristic policy, is determined by a predefined piecewise

(45) $$\rho_{n}=\left\{\begin{array}{cc} 0.82, & \eta_{n}>0.08,\\ 0.58, & 0.02<\eta_{n}\leq 0.08,\\ 0.30, & -0.04<\eta_{n}\leq 0.02,\\ 0.12, & \eta_{n}\leq -0.04. \end{array}\right.$$

Accordingly, the heuristic transmit power and its normalized action are given by

(46) $$P_{n}^{h}=\rho_{n}P_{\max},\;a_{p,h}\left[n\right]=2\rho_{n}-1.$$

For IRS phase design, the heuristic policy aligns the reflected components toward Bob. The phase shift in the *m*-th IRS element is determined according to

(47) $$\theta_{m,h}\left[n\right]={\left[-∠\left(h_{I,b,m}{\left[n\right]}^{*}h_{u,I,m}\left[n\right]\right)\right]}_{2\pi },$$

where $h_{u,I,m}\left[n\right]$ and $h_{I,b,m}\left[n\right]$ denote the UAV-to-IRS and IRS-to-Bob channel coefficients of the m-th IRS element at time slot n, respectively. The corresponding normalized phase action is $a_{\theta ,m,h}\left[n\right]=\frac{\theta_{m,h}\left[n\right]}{\pi }-1$. The heuristic rules provide informative exploration actions while satisfying the corresponding control constraints. They are used only for replay-buffer initialization and exploration guidance, whereas the final control policy is learned by the SAC agent through environment interaction and policy updates.

To enhance initial learning stability and improve sample efficiency, heuristic-generated transitions are used to pre-fill the replay buffer:

(48) $$\begin{array}{c} D_{h}={\left\{\left(s\left[i\right],a_{h}\left[i\right],r\left[i\right],s\left[i+1\right]\right)\right\}}_{i=1}^{N_{h}}, \end{array}$$

where $N_{h}$ denotes the number of heuristic-generated samples collected by interacting with the environment under the heuristic policy. Meanwhile, the actor network is pre-trained on $D_{h}$ to obtain a more stable initial policy before online interaction.

During training, the action is generated by a two-stage hybrid mechanism that combines heuristic guidance and SAC policy learning. During the warm-up stage, for the first $N_{\mathrm{warm}}$ environment interaction steps, the action is exclusively generated from the heuristic policy with additive Gaussian perturbation:

(49) $$a\left[n\right]=\mathrm{clip}\left(a_{h}\left[n\right]+\epsilon \left[n\right],-1,1\right),\;\epsilon \left[n\right]∼N\left(0,\sigma_{g}^{2}\right),$$

where the clipping operation is applied elementwise in the normalized action space. In our implementation, $N_{\mathrm{warm}}=360$ and $\sigma_{g}^{2}=2.25\times 10^{-4}$, corresponding to a standard deviation of $\sigma_{g}=0.015$. After the warm-up stage, the executed action is selected according to

(50) $$a\left[n\right]=\left\{\begin{array}{cc} \mathrm{clip}\left(a_{h}\left[n\right]+\epsilon \left[n\right],-1,1\right),\; & \mathrm{with}\;\mathrm{probability}\;p_{g},\\ a_{\mathrm{SAC}}\left[n\right],\; & \mathrm{with}\;\mathrm{probability}\;1-p_{g}, \end{array}\right.$$

where $a_{h}\left[n\right]$ denotes the heuristic action and $a_{\mathrm{SAC}}\left[n\right]$ denotes the action generated by the SAC actor. The heuristic-guidance probability is fixed at $p_{g}=0.18$. A constant value is adopted to retain a small but persistent contribution from heuristic guidance during training, while allowing the learned SAC policy to dominate with probability $1-p_{g}=0.82$. Therefore, no decay schedule is applied to $p_{g}$ in the current implementation. The clipping operation in (50) only constrains the normalized action components to the interval [−1,1]. After decoding the normalized action into the corresponding physical control variables, the UAV position is further processed by the constraint-enforcing mapping $\Pi_{A_{n}}\left(\cdot \right)$ to satisfy the prescribed flight-region and terminal-reachability constraints, while the transmit power and IRS phase shifts are mapped to their respective feasible ranges. Specifically, let $\tilde{q}\left[n+1\right]={\left[\tilde{x}\left[n+1\right],\tilde{y}\left[n+1\right],H\right]}^{⊤}$ denote the decoded candidate position. The feasible position is obtained through the projection operator

(51) $$\Pi_{A_{n}}\;\left(\tilde{q}\left[n+1\right]\right)=q\left[n+1\right],$$

where $A_{n}=A_{\mathrm{area}}\cap A_{\mathrm{reach},n}$. The projection is implemented sequentially. First, the candidate position $\tilde{q}\left[n+1\right]$ is clipped to the prescribed flight region. Then, if the resulting position violates the terminal-reachability constraint, it is projected onto the boundary of the reachable set. A final area clipping is applied afterward to ensure that the resulting position remains within the prescribed flight region. The speed constraint is enforced beforehand by step-size clipping.

The overall hierarchical HG-SAC optimization procedure is summarized in Algorithm 1.

Since the semantic symbol number $N_{s}$ is a discrete variable, directly incorporating it into the continuous action space of HG-SAC would significantly increase the learning complexity and would complicate the action-space design and increase the learning burden. Therefore, we adopt a hierarchical optimization framework. Specifically, the outer layer searches over candidate semantic symbol numbers, while the inner layer employs the HG-SAC algorithm to optimize the continuous resource allocation variables, including the UAV trajectory, transmit power, and IRS phase shifts. For each candidate $N_{s}$, the corresponding SSEE is obtained from the inner-layer optimization, and the candidate achieving the maximum SSEE is selected. The overall hierarchical HG-SAC optimization procedure is summarized in Algorithm 1.
**Algorithm 1** Hierarchical HG-SAC Algorithm for SSEE Maximization  1:**Initialize:** candidate semantic symbol set $N_{s}=\left\{1,\cdots ,7\right\}$, $N_{s}^{★}$, $\pi^{★}$, $\eta_{\mathrm{SSEE}}^{★}=-\infty$.  2:**for** each candidate semantic symbol number $N_{s}\in N_{s}$ **do**  3:        **Initialize:** actor network $\pi_{\phi }$, twin critic networks $Q_{\omega_{1}}$, $Q_{\omega_{2}}$, and target networks.  4:        Generate heuristic samples according to (41), initialize buffer D.  5:        Pre-train the actor network using heuristic samples.  6:        **for** each training episode **do**  7:                Observe the initial state s[1].  8:                **for** each time slot n=1,⋯,N **do**  9:                       Generate heuristic action $a_{h}\left[n\right]$ according to (41).10:                       Select action a[n] according to the hybrid strategy (50).11:                       Execute action, obtain reward r[n] via (33), observe s[n+1].12:                       Store transition (s[n],a[n],r[n],s[n+1]) into D.13:                       Randomly sample a mini-batch from D.14:                       Compute target *Q*-value according to (37).15:                       Update twin critic networks by minimizing (38).16:                       Update actor network according to (39).17:                       Softly update target networks according to (40).18:                **end for**19:                Decay heuristic guidance probability.20:        **end for**21:        Obtain the optimized policy $\pi_{N_{s}}$.22:        Evaluate the resulting SSEE $\eta_{\mathrm{SSEE}}\left(N_{s}\right)$.23:        **if** $\eta_{\mathrm{SSEE}}\left(N_{s}\right)>\eta_{\mathrm{SSEE}}^{★}$ **then**24:                Update $\eta_{\mathrm{SSEE}}^{★}\leftarrow \eta_{\mathrm{SSEE}}\left(N_{s}\right)$, $N_{s}^{★}\leftarrow N_{s}$, $\pi^{★}\leftarrow \pi_{N_{s}}$.25:        **end if**26:**end for**27:**Output:** Optimal semantic symbol number $N_{s}^{★}$ and optimized policy $\left\{q^{★}\left[n\right],P^{★}\left[n\right],\Theta^{*}\left[n\right]\right\}$.

## 4. Simulation Results and Analysis

### 4.1. Simulation Setup

To evaluate the effectiveness of the proposed joint optimization framework, simulations are conducted in an IRS-assisted UAV semantic secure communication scenario. The UAV flies from the initial position $q_{\mathrm{init}}={\left[0,0,50\right]}^{T}$ to the final position $q_{\mathrm{final}}={\left[100,0,50\right]}^{T}$ within a mission duration of 20 s, while Bob, Eve, and the IRS are located at $q_{B}={\left[72,-45,0\right]}^{T}$, $q_{E}={\left[90,25,0\right]}^{T}$, and $q_{I}={\left[45,0,20\right]}^{T}$, respectively. The communication system operates at a carrier frequency of 3 GHz with a bandwidth of 1 MHz under Rician fading. The semantic transmission performance is evaluated based on the semantic similarity model defined in Section 2.3, which characterizes the joint impact of received SNR and semantic symbol allocation. Unless otherwise specified, the HG-SAC training is conducted within a hierarchical optimization framework, where the semantic symbol number $N_{s}$ is selected from a discrete candidate set and jointly evaluated with the learned continuous resource allocation policy. The UAV propulsion energy follows the rotary-wing energy consumption model under a total energy budget of 4300 J. The remaining system parameters are summarized in Table 1. For the optimization framework, the proposed HG-SAC algorithm is employed to solve the joint optimization of UAV trajectory, transmit power, and IRS phase shifts. The actor and critic networks each consist of two hidden layers with 128 neurons. All learning-related hyperparameters remain fixed across simulations for fair comparison. The simulations are implemented in Python 3.12.7 with PyTorch 2.9.1.

**Figure 4:**
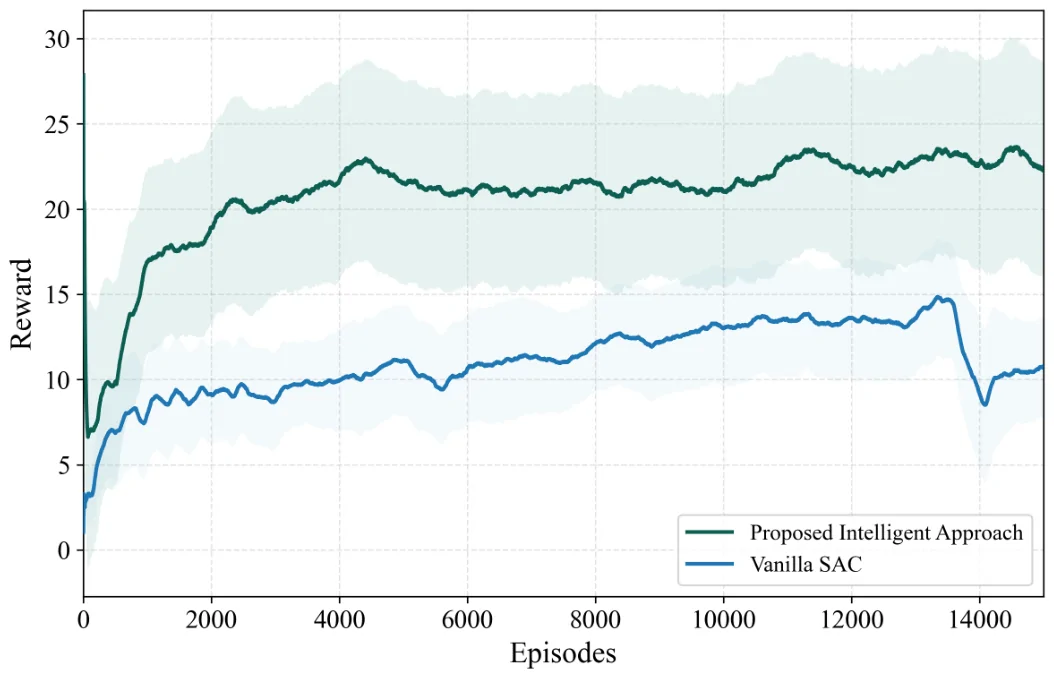
Training reward convergence comparison between the proposed hierarchical HG-SAC algorithm and vanilla SAC.

The training reward in Figure 4 reflects the convergence behavior of the proposed hierarchical HG-SAC algorithm over 15,000 training episodes. The solid curve represents the rolling mean of the training reward, while the shaded band represents the corresponding rolling standard deviation band. For comparison, the convergence curve of vanilla SAC trained under the same simulation settings is also included in Figure 4. Both algorithms gradually improve their policies during training. Compared with vanilla SAC, HG-SAC exhibits a faster increase in training reward during the early training stage and reaches a higher reward level with less training. This indicates that the proposed HG-SAC enhancements facilitate more efficient exploration and accelerate the convergence of the SAC-based learning process. Overall, the convergence comparison demonstrates that the proposed hierarchical HG-SAC framework can effectively learn a stable resource allocation policy for the formulated SSEE maximization problem.

**Figure 5:**
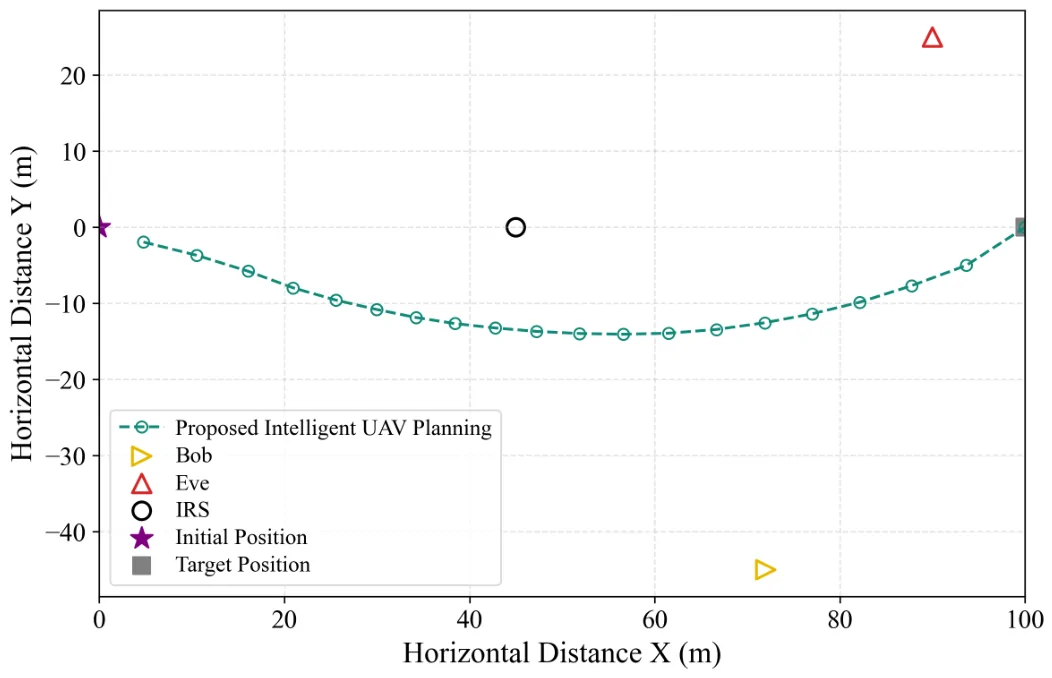
Optimized UAV trajectory learned by the proposed hierarchical HG-SAC algorithm.

The optimized UAV trajectory exhibits clear adaptation to the communication environment, as shown in Figure 5. Instead of following the shortest path between the initial and destination locations, the optimized trajectory is adaptively adjusted according to the proposed SSEE objective. This behavior indicates that the learned policy results in a trajectory that balances communication quality and propulsion energy consumption rather than simply minimizing the flight distance. Specifically, the learned trajectory tends to remain in regions with more favorable communication conditions, thereby improving the potential for secure semantic transmission while satisfying the propulsion energy constraint. In addition, the trajectory evolves smoothly without abrupt directional changes, demonstrating that the learned policy satisfies the UAV mobility constraints while maintaining stable flight dynamics. These observations confirm that the proposed hierarchical HG-SAC framework effectively captures the trade-off among communication performance, secure semantic transmission, and energy efficiency, ultimately leading to improved SSEE performance.

**Figure 6:**
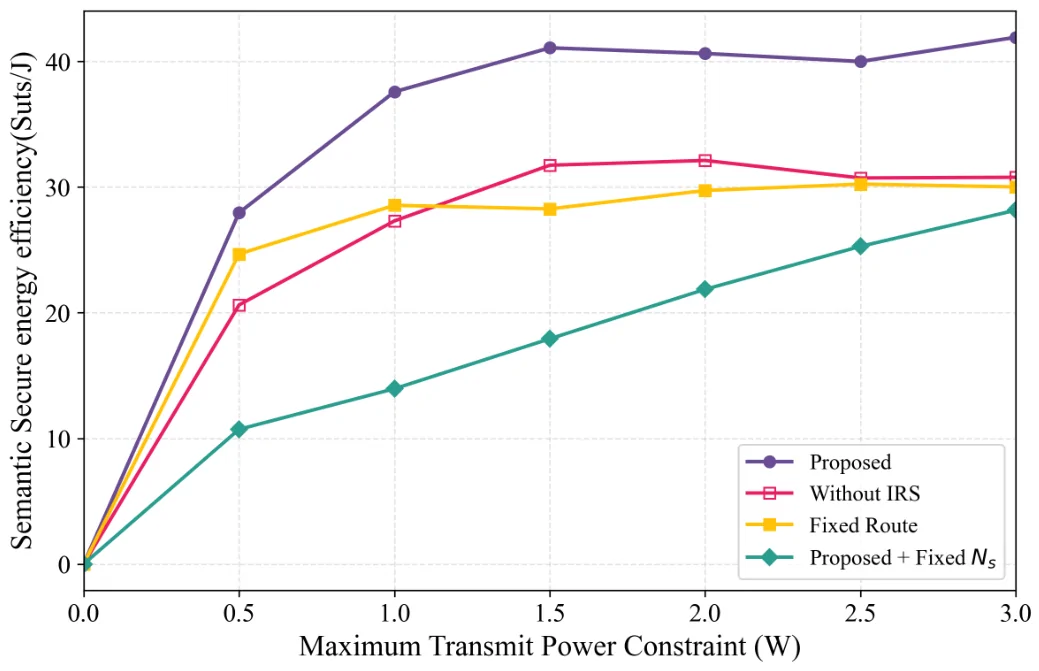
SSEE versus UAV maximum transmit power under different schemes.

Figure 6 reveals the SSEE performance versus the maximum transmit power constraint under different schemes. It can be observed that the SSEE of all schemes increases significantly in the low-power region, since a larger power budget provides more flexibility for enhancing the legitimate semantic transmission capability and increasing the achievable secrecy semantic information. However, as the maximum transmit power constraint further increases, the SSEE gradually approaches a plateau. This is because the improvement in the secrecy semantic information becomes limited once the semantic transmission quality of the legitimate link has been sufficiently enhanced, and additional power resources can only provide marginal performance gains. Among all considered schemes, the proposed joint optimization consistently achieves the highest SSEE over the entire power range. This demonstrates the effectiveness of jointly optimizing UAV trajectory, transmit power, IRS phase shifts, and semantic resource configuration. In contrast, the “Without IRS” scheme suffers from the absence of IRS-enabled passive beamforming gain, while the “Fixed Route” scheme cannot fully exploit UAV mobility to improve the channel conditions. Moreover, the inferior performance of the “Proposed + Fixed $N_{s}$” scheme indicates that adaptive semantic symbol allocation is essential for achieving higher semantic secure energy efficiency.

**Figure 7:**
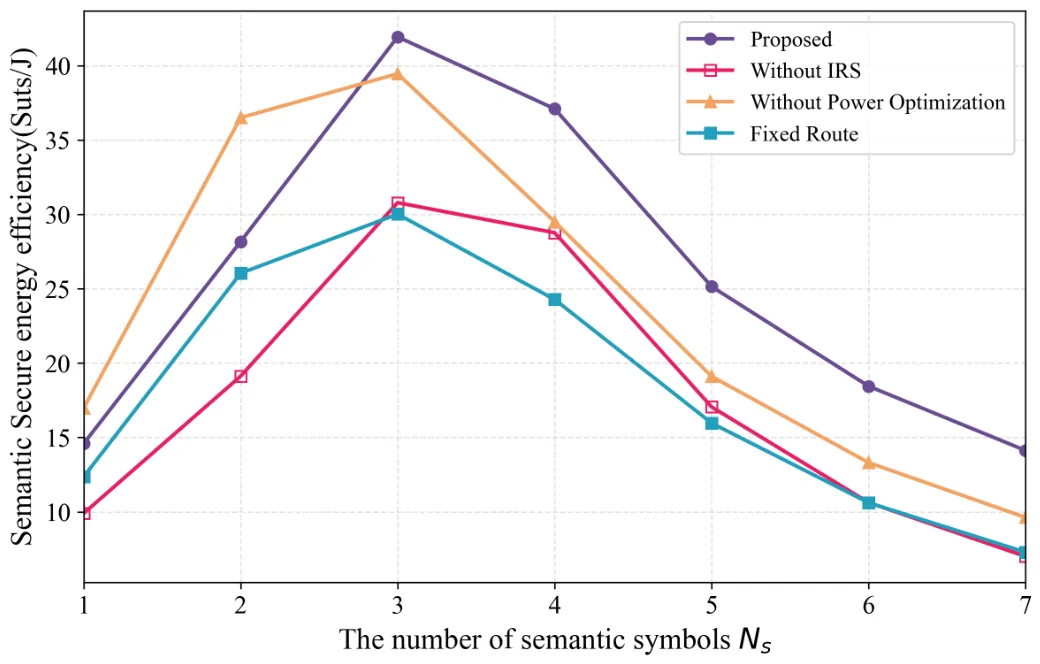
SSEE versus the number of semantic symbols $N_{s}$ under different schemes.

Figure 7 presents the SSEE performance versus the number of semantic symbols $N_{s}$ under different optimization schemes. It can be observed that the SSEE first increases and then decreases with the increase of $N_{s}$, indicating the existence of an optimal semantic symbol allocation. When $N_{s}$ is relatively small, increasing the number of semantic symbols enhances the semantic representation capability and improves the reconstruction quality at the legitimate receiver. Consequently, the secrecy semantic transmission performance is improved, leading to a higher SSEE. However, when $N_{s}$ exceeds a certain range, the SSEE gradually decreases. This is because the improvement in semantic representation capability becomes marginal after the semantic information is sufficiently captured. As a result, further increasing $N_{s}$ cannot provide proportional enhancement in the secrecy semantic transmission performance, leading to a decrease in SSEE. Therefore, an appropriate selection of $N_{s}$ is essential to balance semantic representation capability and transmission efficiency. In particular, the proposed joint optimization scheme achieves its maximum SSEE of 41.93 Suts/J at $N_{s}=3$. At this operating point, it outperforms the “Without IRS”, “Without Power Optimization”, and “Fixed Route” schemes by 36.2%, 6.28%, and 39.7%, respectively. These quantitative results demonstrate the effectiveness of jointly optimizing the UAV trajectory, transmit power, and IRS phase shifts for SSEE maximization.

It can be observed that the optimal semantic-symbol number remains stable with respect to the IRS size over the tested range, where the optimum is consistently attained at $N_{s}=3$ for all tested IRS configurations, as summarized in Table 2. In contrast, the optimum under different SNR settings varies within a relatively narrow range. Specifically, $N_{s}=3$ is optimal at the baseline SNR setting (0 dB offset) and also remains optimal under a moderately improved SNR condition (+3 dB offset), while the optimum shows a tendency toward smaller values under the higher-SNR conditions considered here and toward larger values under the lower-SNR conditions considered here. This behavior is consistent with the inherent tradeoff of semantic transmission. Under the higher-SNR conditions considered here, reliable semantic reconstruction can be achieved with fewer semantic symbols, whereas the lower-SNR cases considered here favor a relatively larger $N_{s}$, which may provide additional robustness for semantic information recovery.

**Figure 8:**
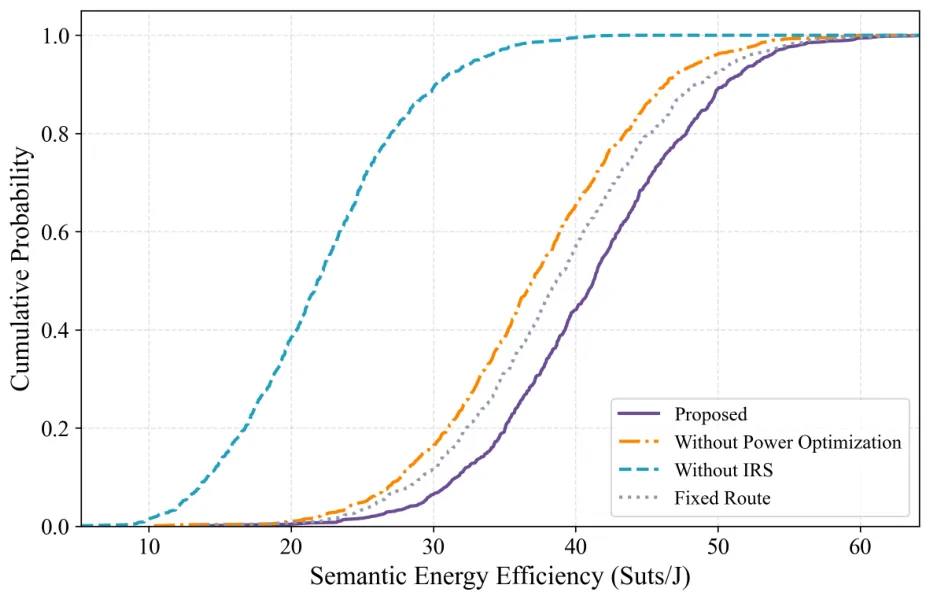
Cumulative distribution function of the SSEE under different schemes.

The cumulative distribution function (CDF) curves in Figure 8 highlight the distributional advantage of the proposed joint optimization scheme over the benchmark schemes. It can be observed that the CDF curve of the proposed joint optimization scheme is shifted toward higher SSEE values compared with those of the benchmark schemes. This indicates that the proposed framework shifts the SSEE distribution toward higher values rather than merely improving the average SSEE. In particular, the proposed scheme attains a median SSEE of 41.26 Suts/J, yielding gains of 11.5%, 6.8%, and 88.7% over the “Without Power Optimization”, “Fixed Route”, and “Without IRS” schemes, respectively. These quantitative results demonstrate that the proposed framework achieves higher SSEE values across a broad range of channel realizations. Moreover, the proposed framework has a lower probability of obtaining low SSEE values, indicating its capability of maintaining stable semantic secure energy efficiency under different channel conditions. This performance improvement is attributed to the joint optimization of UAV trajectory, transmit power allocation, IRS phase shifts, and semantic resource configuration, enabling the system to achieve improved secure semantic transmission performance. Overall, the CDF results further validate the effectiveness and robustness of the proposed optimization framework for maximizing semantic secure energy efficiency.

**Figure 9:**
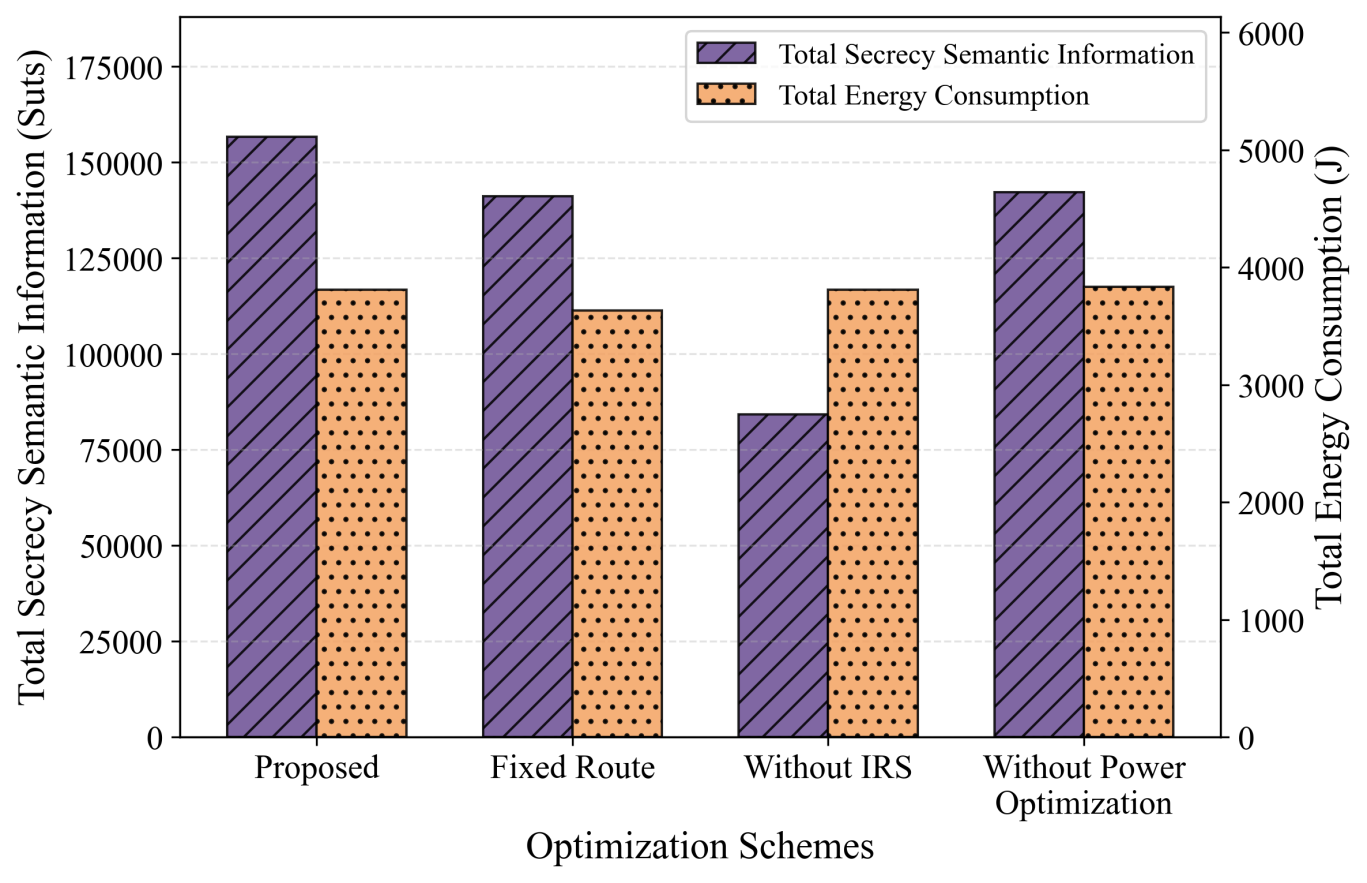
Trade-off between Total Secrecy Semantic Information and Energy Consumption under Different Schemes.

The proposed joint optimization achieves the most favorable trade-off between total secrecy semantic information and energy consumption among the considered schemes, as shown in Figure 9. In particular, the proposed scheme achieves $1.57\times 10^{5}$ Suts of total secrecy semantic information, which is 11.0%, 86.0%, and 10.1% higher than the “Fixed Route”, “Without IRS”, and “Without Power Optimization” schemes, respectively, while maintaining a comparable total energy consumption of 3807 J. These quantitative results demonstrate that the proposed framework can obtain substantially higher secrecy semantic information without incurring a significant increase in energy consumption. The performance improvement is attributed to the joint optimization of UAV trajectory, transmit power allocation, IRS phase shifts, and semantic resource configuration, which enables more effective utilization of the available energy for secure semantic transmission. Overall, these results demonstrate that the proposed framework achieves a better trade-off between secrecy semantic information and total energy consumption, further validating the effectiveness of the proposed SSEE-oriented optimization framework.

**Figure 10:**
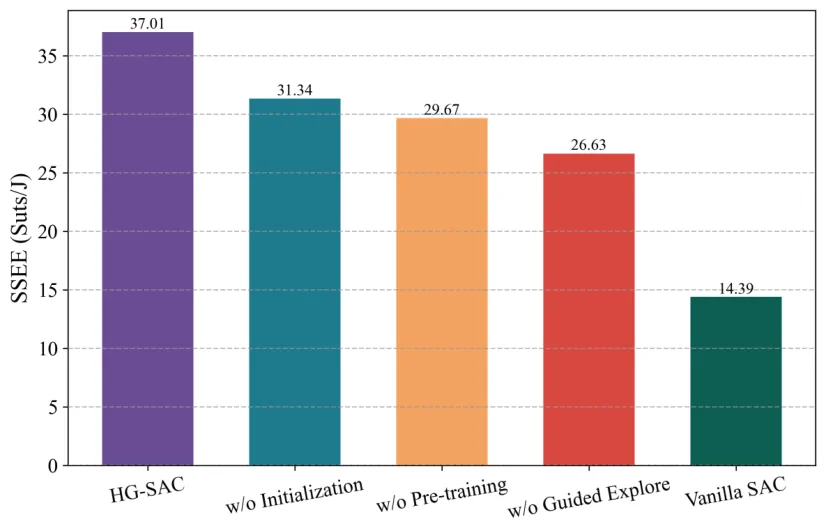
Ablation study of the individual components of the proposed HG-SAC framework.

To further investigate the contributions of the individual components in the proposed HG-SAC framework, an ablation study is conducted by separately removing each of the initialization, policy pre-training, and heuristic-guided exploration mechanisms. The resulting SSEE performance is presented in Figure 10. As shown in Figure 10, the complete HG-SAC achieves the highest SSEE of 37.01 Suts/J. Removing the initialization, policy pre-training, and heuristic-guided exploration reduces the SSEE to 31.34, 29.67, and 26.63 Suts/J, corresponding to decreases of 15.32%, 19.83%, and 28.05%, respectively. Among the three components, heuristic-guided exploration has the largest impact on the final SSEE performance. In comparison, Vanilla SAC achieves only 14.39 Suts/J, which is 61.12% lower than HG-SAC. These results indicate that all three components contribute positively to the overall performance of HG-SAC.

### 4.2. Sensitivity Analysis with Respect to Key System Parameters

**Figure 11:**
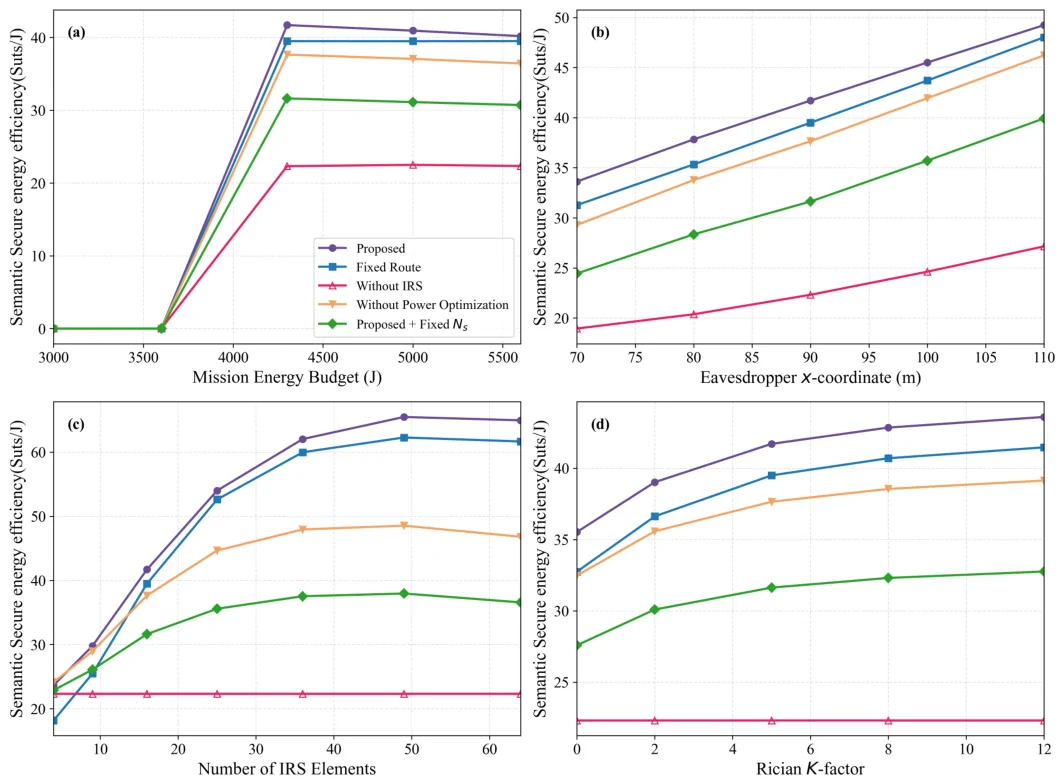
Sensitivity analysis of SSEE under different mission energy budgets, eavesdropper positions, IRS sizes, and Rician *K*-factors: (**a**) mission energy budget; (**b**) eavesdropper *x*-coordinate; (**c**) number of IRS elements; (**d**) Rician *K*-factor.

To further validate the robustness of the proposed method, we investigate its SSEE performance under different mission energy budgets, eavesdropper positions, IRS sizes, and Rician *K*-factors. The corresponding results are shown in Figure 11. Overall, these results mainly serve to verify that the proposed design exhibits physically consistent performance trends and maintains its performance advantage under different resource, geometric, IRS configuration conditions, and channel conditions.

As shown in Figure 11a, a clear feasibility threshold can be observed with respect to the mission energy budget. When the budget is 3000 J or 3600 J, all schemes yield zero effective SSEE under the adopted strict feasibility criterion. Once the budget reaches 4300 J, all schemes become feasible and their SSEE rises sharply. For example, at 4300 J, the proposed method achieves about 41.7 Suts/J, which is higher than the fixed-route scheme (about 39.5 Suts/J), the scheme without power optimization (about 37.8 Suts/J), and the scheme without IRS (about 22.3 Suts/J). Beyond this point, the curves remain relatively stable, suggesting that further increasing the energy budget provides limited additional SSEE improvement under the considered settings. Figure 11b shows that the SSEE of all schemes increases monotonically as the eavesdropper moves farther away along the *x*-axis. This behavior is mainly attributed to the increased distance between Eve and the transmission region, which weakens the eavesdropping channel relative to the legitimate link. The proposed method remains consistently superior, increasing from about 33.7 Suts/J at $x_{E}=70$ m to about 49.2 Suts/J at $x_{E}=110$ m, which demonstrates robust performance across the tested eavesdropper locations. As illustrated in Figure 11c, increasing the number of IRS elements significantly improves the SSEE of all IRS-enabled schemes, whereas the scheme without IRS remains nearly unchanged at about 22.3 Suts/J. This confirms that the performance improvement is primarily attributable to the additional passive beamforming capability provided by the IRS. For the proposed method, the SSEE rises from about 23.3 Suts/J at M=4 to about 65.0 Suts/J at M=49, and then becomes nearly saturated, which indicates diminishing returns for very large IRS sizes. Figure 11d further shows that a larger Rician *K*-factor leads to higher SSEE for the IRS-enabled schemes, because a larger Rician *K*-factor corresponds to a stronger LoS component relative to the scattered component, which provide more favorable and predictable propagation conditions for IRS-assisted beamforming. The proposed method consistently achieves the best performance, increasing from about 35.6 Suts/J at K=0 to about 43.3 Suts/J at K=12, while the scheme without IRS remains almost unchanged.

### 4.3. Robustness to Imperfect CSI

**Figure 12:**
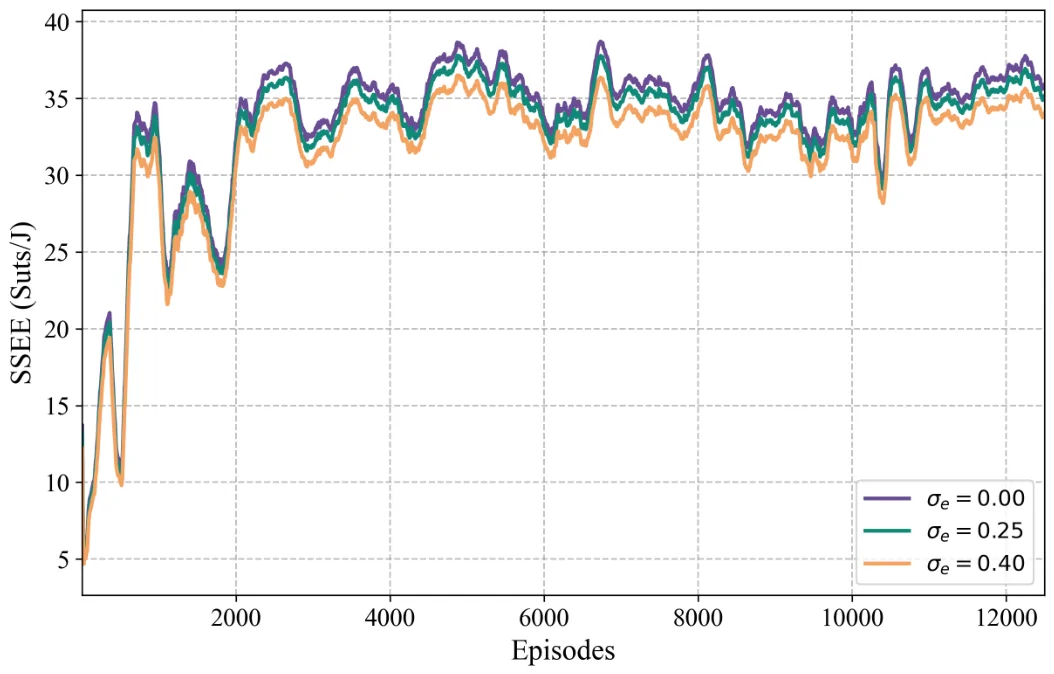
Validation SSEE versus training episodes under different levels of CSI estimation error.

To further examine the robustness of the proposed HG-SAC framework under practical channel estimation conditions, additional simulations are conducted by considering imperfect channel state information (CSI). Specifically, the estimated channel is modeled as

(52) $$\hat{h}=h+e,$$

where h denotes the actual channel, $\hat{h}$ is the estimated channel, and e represents the CSI estimation error. In the simulations, the error severity is controlled by the standard deviation parameter $\sigma_{e}$, where $\sigma_{e}=0$ corresponds to the perfect-CSI case considered in the main results. The estimated CSI is introduced in the environment-side channel computation rather than being directly observed by the HG-SAC policy, while the achieved performance is evaluated using the true channel realizations. Figure 12 plots the validation SSEE versus training episodes under different CSI estimation error levels. It can be observed that the proposed HG-SAC framework remains stable and convergent under all considered error levels. As $\sigma_{e}$ increases, the validation SSEE gradually decreases and exhibits slightly larger fluctuations, since more severe CSI uncertainty makes channel-dependent secure semantic transmission more difficult. Nevertheless, the learning process remains well behaved, indicating that the proposed method preserves satisfactory robustness under imperfect CSI.

**Figure 13:**
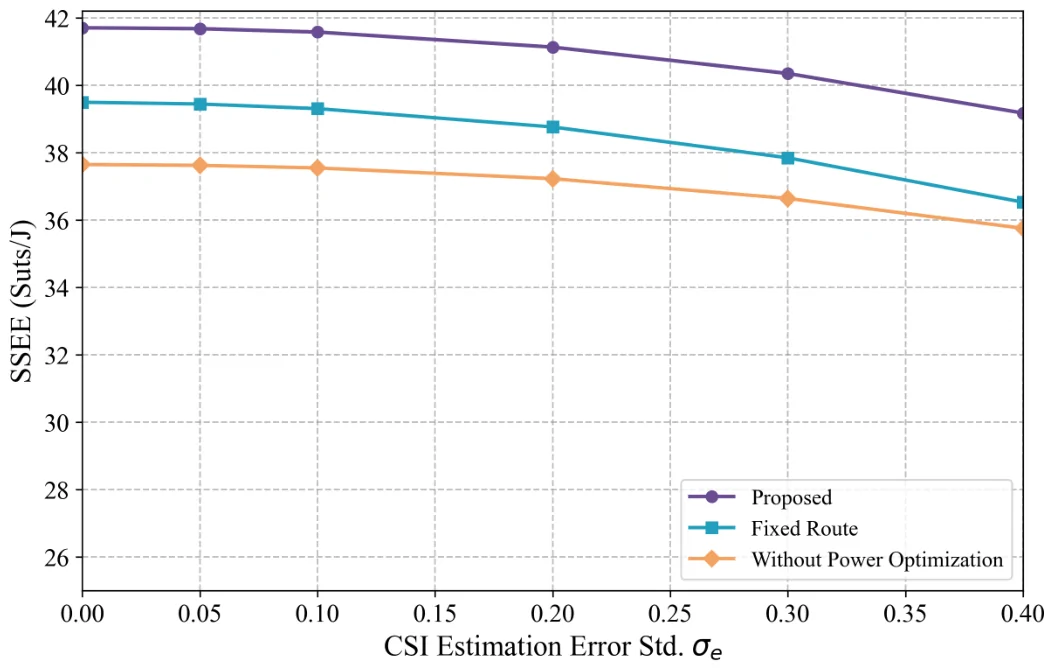
SSEE versus CSI estimation error standard deviation.

Figure 13 further illustrates the impact of CSI estimation errors on the achieved SSEE. As the estimation error level increases, the SSEE gradually decreases because inaccurate CSI reduces the effectiveness of channel-aware transmission design. However, the degradation remains gradual over the considered range, which demonstrates that the proposed framework maintains reasonable robustness against CSI imperfections, while the perfect-CSI assumption serves as an optimistic upper-bound benchmark.

## 5. Conclusions

This paper investigates an IRS-assisted UAV semantic secure communication system to maximize the SSEE in the presence of an Eve. We jointly design the UAV trajectory, transmit power, and IRS phase shifts under both secrecy and energy constraints. A hierarchical framework based on the HG-SAC algorithm handles the continuous resource allocation, while the influence of the number of semantic symbols is examined to determine a suitable semantic transmission configuration. Simulation results are assessed in terms of SSEE performance curves, cumulative distribution function, optimized UAV trajectory, and the trade-off between secrecy semantic information and energy consumption. Compared with several benchmark schemes, including the cases without IRS, with fixed power allocation, a fixed route, and fixed semantic symbol assignment, the proposed approach achieves higher SSEE. The results also highlight that choosing an appropriate number of semantic symbols is key to improving SSEE. The optimized UAV trajectory satisfies the mobility constraints while maintaining a smooth flight path without abrupt direction changes. In summary, by jointly leveraging IRS, UAV mobility, and semantic communication, the proposed framework provides an effective means to enhance SSEE in eavesdropping environments.

Despite these advantages, several limitations remain. Although the impact of channel estimation errors has been evaluated, more comprehensive CSI uncertainty models and robust designs remain to be investigated. In addition, the current framework assumes ideal IRS operation and considers static Bob/Eve locations, a fixed UAV altitude, and a lookup-table-based characterization of semantic performance. Future work will investigate more practical designs under non-ideal IRS hardware and more comprehensive CSI uncertainty models, as well as dynamic user/eavesdropper scenarios, three-dimensional UAV trajectory optimization with variable altitude, and more flexible semantic performance modeling beyond lookup tables.

## Figures and Tables

**Table 1:** Simulation Parameter Settings.

Parameter	Value
Carrier frequency $f_{c}$	3 GHz
Bandwidth *W*	1 MHz
Maximum transmit power $P_{\max}$	3 W
Path loss exponent α	2.8
Path loss exponent β	2.2
Rician factor *K*	5
IRS elements *M*	16
UAV altitude $H_{0}$	50 m
Maximum speed $V_{\max}$	15 m/s
Flight duration *T*	20 s
Time slots *N*	20
Semantic symbol number $N_{s}$	{1,2,…,7}
Sentence length *L*	12
Blade profile power $P_{0}$	79.86 W
Induced power $P_{i}$	88.63 W
Energy budget $E_{\max}$	4300 J
Discount factor γ	0.99
Actor learning rate	$3\times 10^{-4}$
Critic learning rate	$3\times 10^{-4}$
Temperature learning rate	$3\times 10^{-4}$
Batch size	64
Number of heuristic-generated samples $N_{h}$	320
Replay-buffer capacity	$10^{5}$
Soft target-update coefficient τ	0.005
Entropy temperature α	Automatically tuning, $\alpha_{0}=1$

**Table 2:** Optimal semantic-symbol number $N_{s}$ under different SNR and IRS-size settings.

Setting	Optimal $N_{s}^{★}$	Peak SSEE (Suts/J)
SNR offset =−6 dB	5	33.01
SNR offset =−3 dB	4	40.94
SNR offset =0 dB	3	41.93
SNR offset =+3 dB	3	41.24
SNR offset =+6 dB	2	40.32
IRS size M=4	3	23.65
IRS size M=12	3	34.87
IRS size M=16	3	41.70
IRS size M=24	3	53.50

## Data Availability

The simulation data and code presented in this study are available upon reasonable request from the corresponding author.
